# Characterization of Key Odorants in Lingtou Dancong Oolong Tea and Their Differences Induced by Environmental Conditions from Different Altitudes

**DOI:** 10.3390/metabo12111063

**Published:** 2022-11-03

**Authors:** Miao Wang, Jianlong Li, Xiaohui Liu, Chengshun Liu, Jiajia Qian, Jie Yang, Xiaochen Zhou, Yongxia Jia, Jinchi Tang, Lanting Zeng

**Affiliations:** 1 Guangdong Provincial Key Laboratory of Applied Botany, Key Laboratory of South China Agricultural Plant Molecular Analysis and Genetic Improvement, South China Botanical Garden, Chinese Academy of Sciences, No. 723 Xingke Road, Guangzhou 510650, China; 2 University of Chinese Academy of Sciences, Beijing 100049, China; 3 South China National Botanical Garden, No. 723 Xingke Road, Guangzhou 510650, China; 4 Tea Research Institute, Guangdong Academy of Agricultural Sciences, Guangdong Provincial Key Laboratory of Tea Plant Resources Innovation and Utilization, Guangzhou 510640, China; 5 College of Tea Science, Yunnan Agricultural University, Kunming 650201, China; 6 Center of Economic Botany, Core Botanical Gardens, Chinese Academy of Sciences, Guangzhou 510650, China

**Keywords:** Lingtou Dancong, stir-bar sorptive extraction, gas chromatography-olfactometry/mass spectrometry, odorants, different altitudes

## Abstract

Lingtou Dancong oolong tea is a famous Chinese oolong tea due to its special honey-like aroma. However, little is known about its specific aroma profile and key contributors. Furthermore, whether the aroma characteristics of Lingtou Dancong oolong tea are affected by the environmental conditions at different altitudes is unknown. In this study, the aromas in Lingtou Dancong oolong tea were extracted and analyzed by stir-bar sorptive extraction (SBSE) combined with gas chromatography-olfactometry (GC-O) and GC-mass spectrometry (GC-MS), and the aroma profiles of tea plants grown at different altitudes were compared. We detected 59 odor compounds in Lingtou Dancong oolong tea. Eight compounds with honey and floral odors were identified as key components on the basis of GC-O, GC-MS, odor activity value, and flavor dilution analyses. Differences in the contents of precursor geranyl diphosphate and transcript levels of structural genes were found to be responsible for the differential accumulation of linalool and hotrienol among plants grown at different altitudes. This is the first report on the aroma characteristics and key contributors of Lingtou Dancong oolong tea and their differences, as affected by altitude. These results provide details of the chemical basis of the aroma quality of Lingtou Dancong oolong tea.

## 1. Introduction

Tea is one of the most popular non-alcoholic beverages in the world and its popularity is closely related to its delicious taste and pleasant aroma [1]. Different processing methods contribute to taste and aroma. Tea can be divided into six categories, according to its quality characteristics and processing methods: green tea, white tea, yellow tea, oolong tea, black tea, and dark tea. Among them, oolong is semi-fermented tea, and therefore has both the fragrance of green tea and the mellow taste of black tea. It is also famous for its rich and varied floral fragrance. Dancong tea, which is mainly produced in the Guangdong province, has a long history of cultivation in China. This type of tea includes *Camellia sinensis* cv. Lingtou Dancong, a national variety in China, whose core production area is the Fenghuang mountain. Oolong tea made from fresh leaves of *C. sinensis* cv. Lingtou Dancong is loved by consumers because of its unique honey-like flavor. It has been approved as a product with high economic value and a national geographical indication.

The aroma of oolong tea is affected by many factors before and after harvesting. At the pre-harvest stage, the genotype of the tea variety plays an important role in the aroma of tea products, and the aroma of Dancong tea produced from fresh leaves differs widely among varieties and strains [1,2,3]. The growing environment of tea plants is another crucial factor in aroma formation. The ecological environment as a whole (i.e., the combination of light, temperature, and soil conditions) at different altitudes affects the types and contents of aroma compounds in Dancong tea [4]. The turnover process of oolong tea also affects its quality. However, few studies have explored the unique aroma characteristics of tea products made from the Lingtou Dancong cultivar (also known as Baiye Dancong), and whether this special aroma is affected by environmental factors [2].

Aroma compounds can be extracted using a variety of methods, including solvent extraction and headspace collection, and detected using methods such as gas chromatography-mass spectrometry (GC-MS) and GC-olfactometry (GC-O). These detection methods can be supplemented by statistical analyses and various calculations to analyze aroma compounds in detail [5]. More than 700 aroma compounds have been identified in tea leaves and tea soups, but the aroma characteristics of tea leaves are determined by a few key contributing aroma compounds. Many studies have shown that the human-perceivable odor is related to the threshold and concentration of aroma compounds, so the information obtained by a detector cannot completely reflect the real aroma [5]. Therefore, GC-O/MS is a promising method for fragrance analyses.

In this study, the aroma components in leaves of Lingtou Dancong oolong tea plants grown at different altitudes were comprehensively analyzed by GC-O/MS. The aroma components of oolong tea grown at different altitudes were identified and quantified, and the reasons for differences in their types and contents among these materials were explored. The overall aim of this study was to determine which compounds are responsible for the honey-like aroma of Lingtou Dancong oolong tea and to provide specific guidance for cultivating tea under the most suitable environmental conditions.

## 2. Materials and Methods

### 2.1. Materials and Treatments

The oolong tea was made from *C. sinensis cv*. Lingtou Dancong picked from Danhu village, Fenghuang town, Chaoan district (810 m), Qipan village, Fenghuang town, Chaoan district (560 m), and Xiaxiao village, Fubin town, Raoping distinct (250 m) in Chaozhou, Guangdong, China. All the materials were harvested in April 2020 and were evaluated using the stir-bar sorptive extraction (SBSE) approach combined with a gas chromatography-olfactometry/mass spectrometry (GC-O/MS) technique. The picking standard was one bud, two or three leaves, and the stem. The picked fresh leaves were made into oolong tea [1]. The processing method was as follows: the freshly plucked tea leaves were exposed to sunlight, outside, for 25–40 min, and then indoor-withered at 30 °C until the humidity of the leaves fell to 70%. The tea leaves were turned over 5–7 times every 1.5–2 h, rolled and dried at 120–130 °C for 10 min. Finally, the tea leaves were dried at 85–90 °C for 3 h.

The fresh leaves of *C. sinensis* cv. Lingtou Dancong were picked from plants cultivated at different altitudes in Chaozhou, Guangdong, China. The picking altitudes were 730 m (high altitude), 380 m (medium altitude), and 110 m (low altitude). All samples were harvested in October 2021, and the picking standard was one bud, two or three leaves, and the stem or clip leaf. Wounding stress from the oolong tea manufacturing process was the key factor responsible for the aroma formation [1]. Therefore, a portion of the picked leaves was subjected to a single wounding treatment (standing for 3 h) and another portion was subjected to a continuous wounding treatment (shaking for 3 h at 300 rpm). The temperature of both the single wounding treatment and continuous wounding treatment were 26 °C and the humidity were 70%. All samples were quickly frozen in liquid nitrogen and then stored at −80 °C until further analysis.

### 2.2. Extraction of Volatile Compounds

Volatile compounds were extracted by SBSE, as described elsewhere [6]. Each ground tea sample (1 g) was weighed into a headspace glass bottle (Agilent Technologies, Palo Alto, CA, USA). A polydimethylsiloxane twister and 10 mL distilled water, heated to 100 °C (Hangzhou Wahaha Group Co., Ltd., Hangzhou, China), were quickly added into the bottle, the bottle was tightly capped, and then the contents were stirred at 70 °C for 30 min at 1000 rpm. Finally, the volatiles were analyzed using a thermal desorption unit-cooled injection system (TDU-CIS) and GC-MS. The parameters for TDU-CIS, GC-MS, and GC-O were as described in a previous study [7]. The temperature program for the TDU was as follows: initial temperature of 30 °C (maintained for 1 min), increased to 240 °C in 2.4 min, and maintained for 5 min. The temperature program for the CIS-4 (Gerstel) GC inlet was −100 °C for 1 min, increased to 280 °C at 12 °C/s, and then held for 3 min. An Agilent 7890B/5977B GC equipped with an HP-5MS column (30 m × 250 μm × 0.25 μm, Agilent) was used to detect and quantify odor compounds using the parameters described previously, with some changes [6]. The temperature of quadrupole and mass spectrometer transmission line were set to 150 °C and 250 °C, respectively. The full scan range was *m/z* 40–450.

### 2.3. Identification and Quantification of Aroma Compounds in Lingtou Dancong Oolong Tea

The improved standard addition method was used to identify odorants [8]. A series of mixed standards was added to the prepared blank matrix of tea with the aroma compounds removed. The blank matrix was extracted and analyzed by the method described in Section 2.2. The peak area of odorants in samples was substituted into the standard curve to calculate their contents accurately. Methyl salicylate, geraniol, indole, and *trans*-β-ionone were ordered from Aladdin Corp. (Shanghai, China). α-Ionone, γ-nonanolactone, β-myrcene, 1-octen-3-ol, nonanal, butanal, 2-methyl-, dihydroactindiolide, jasmine lactone, γ-octanolactone, (*E*)-2-octenal and other chemicals were purchased from J&K Scientific Ltd. (Beijing, China) or Sigma-Aldrich (Shanghai, China).

The aroma compounds were analyzed using the Agilent ChemStation/LeCo Chroma TOF workstation and identified by comparison with data in the NIST2014 library, n-alkane retention indices (C8–C40), and authentic standards, by using tools in ODI software (Gerstel EL GMBH & Co., Mülheim An Der Ruhr, Germany), and on the basis of aroma standard descriptions (http://www.thegoodscentscompany.com/search3.php?qOdor=20126-76-5&submit.x=9&submit.y=9, accessed on 26 August 2022). Accurate quantification was performed by authentic standards. For available standards, the authentic standard was used for quantification. Some standards were not available, so the quantitative analysis was carried out by standards with similar structures, which are listed in Table 1.

### 2.4. GC-O Analysis

Aroma compounds were analyzed using a GC-MS instrument equipped with an ODP-3 olfactory detection port (ODP; Gerstel GmbH & Co. KG, Mülheim an der Ruhr, Germany), using the parameters described elsewhere [6]. The GC-O analysis group consisted of six healthy assessors with extensive evaluation experience [7]. All assessors were well trained for GC-O, having received at least 30 h of training with standard samples and tea samples. The assessors were trained to identify and determine the strength of the standards, as well as to be professionally trained in the sensory evaluation of tea. The sensory assessors described the odor properties of the compounds, and also the odor intensity. The aroma intensity was scored on a scale of 1 to 4, where “1” indicated the weakest aroma intensity, “2” indicated moderate aroma intensity, “3” indicated strong aroma intensity, and “4” indicated the strongest aroma intensity [9]. The flavor dilution (FD) analysis was completed by three highly trained assessors. The FD factor of the compound was determined by adjusting the split ratio (16:1; 32:1; 64:1; 128:1; 256:1; 512:1) to dilute the aroma extract [7]. The dilution multiple corresponding to the lowest concentration that the reviewers could smell was the FD value (16, 32, 64, 128, 256, 512) of that compound.

### 2.5. Calculation of Odor Activity Value

The odor activity value (OAV) describes the contribution of individual aroma components to the overall aroma; it is the ratio of the content of a compound to its threshold in water, and OAV > 1 is considered to indicate a significant contribution [7]. The contents of the volatiles were determined by GC-MS and information about thresholds was obtained from the literature (Table S1).

### 2.6. Extraction of Linalool from Fresh Leaves

Linalool was extracted and analyzed by GC-MS using the parameters described in our previous study [10]. A 300-mg (fresh weight) portion of powdered sample was extracted overnight with shaking at room temperature with dichloromethane (2.7 mL), containing ethyl decanoate (0.5 nmol) as the internal standard. The resulting liquid was dried and concentrated by anhydrous Na_2_SO_4_ under a nitrogen stream, and then analyzed using a QP2010SE (Shimadzu Corporation, Kyoto, Japan) GC-MS instrument, equipped with a Supelcowax-10 column (30 m × 250 μm × 0.25 μm, Supelco Inc., Bellefonte, PA, USA). Linalool was identified by comparison with the authentic standard.

### 2.7. Extraction of Geranyl Diphosphate from Fresh Leaves

Geranyl diphosphate (GPP) was extracted from fresh leaves and analyzed as described in our previous study [10]. A 200-mg portion of powdered fresh sample was mixed with 2 mL GPP buffer (50 mM Tris/HCl, pH 8.0, 20 mM DTT, 20 mM MgCl_2_, and 5% glycerin) and 20 μL 1 M sodium molybdate, then the mixture was vortexed, ultrasonically extracted in ice-cold water, and centrifuged (6000× *g*, 4 °C, 10 min). The supernatant was extracted with hexane to remove volatiles. The upper phase was collected and hydrolyzed using 2 mL 1 M H_2_SO_4_ at 37 °C for 30 min to release linalool. The mixture was kept on ice for 15 min, before adding 100 ng [^2^H_3_]linalool and 100 mg NaCl. The solution was extracted with hexane: ethyl acetate (1:1) and then concentrated by drying before GC-MS analysis. The analysis parameters were as described in Section 2.6, with some changes. The full-scan mode was used to detect linalool and [^2^H_3_]linalool, and the linalool content was calculated using data collected in the SIM mode (*m/z* = 71, 74, 93, 96).

### 2.8. Analysis of Gene Transcript Levels

The extraction of RNA and the reverse transcription to generate cDNA were as described in our previous study [10]. The primers used for qRT-PCR are shown in Table S2. The thermal cycling program was as follows: 95 °C for 30 s, followed by 40 cycles of 95 °C for 5 s and 60 °C for 40 s on qTOWER384/G instrument (Analytik, Jena, Germany). The 2^−ΔΔct^ method was used to calculate relative gene transcript levels. The reference genes were encoding Elongation Factor1 (*CsEF-1α*) and SAND family protein (*CsSAND*).

### 2.9. Statistical Analysis

Statistical analyses were performed using SPSS software (Ver25.0, SPSS Inc., Chicago, IL, USA). Differences among three or more groups were determined using one-way analysis of variance followed by Duncan’s multiple comparison tests. Differences were considered significant at *p* ≤ 0.05. The principal component analysis (PCA) and orthogonal partial least squares-discriminant analysis (OPLS-DA) were conducted using SIMCA V14.1 (Umetrics AB, Umea, Sweden). An internal seven-fold cross-validation and 200 random permutation tests were conducted to verify the model.

## 3. Results

### 3.1. Differences in Aroma Composition of Lingtou Dancong Oolong Tea among Different Altitudes

**Figure 1:**
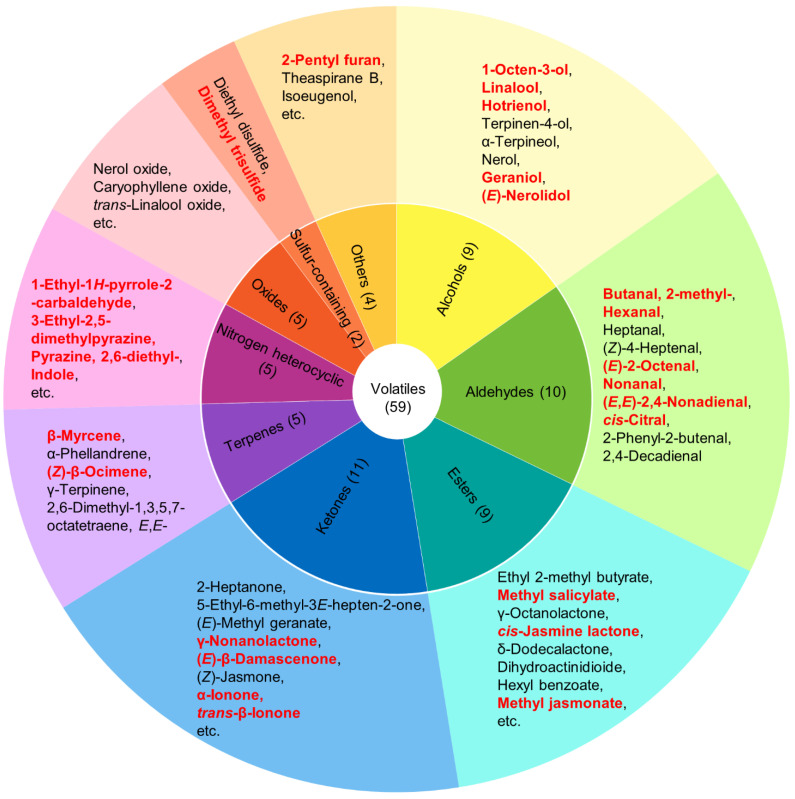
Categories of aroma compounds and specific compounds in Lingtou Dancong oolong tea product. The number in brackets indicates the number of compounds in each category. Compounds with an odor activity value (OAV) higher than 1 are highlighted in red.

**Figure 2:**
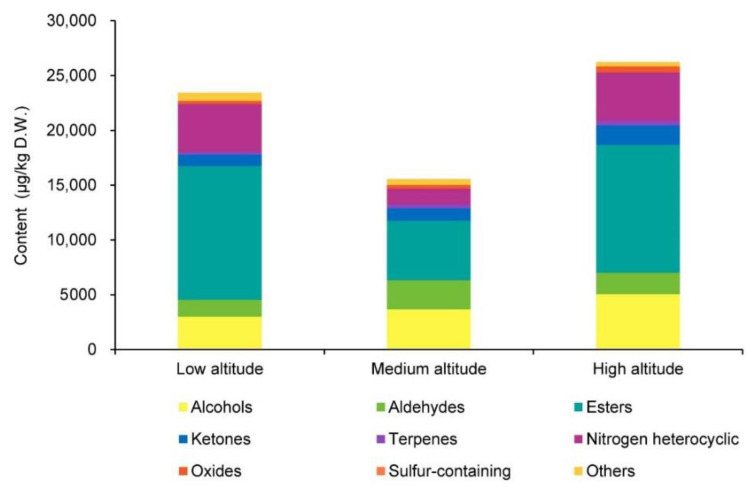
Categories of aroma compounds and total aroma contents in Lingtou Dancong oolong tea product from different altitudes. High altitude, medium altitude and low altitude represent the Lingtou Dancong oolong teas from high, medium and low altitude, respectively. D.W. represents dry weight.

We detected 59 volatile compounds common to the Lingtou Dancong oolong teas produced from plants grown at three different altitudes (Table 1). These compounds were classified into nine categories: aldehydes, alcohols, esters, ketones, terpenoids, oxides, nitrogen heterocyclic compounds, sulfur-containing compounds, and others (Table 1). The most abundant compounds were ketones (11), followed by aldehydes (10), alcohols (9), and esters (9) (Figure 1). Esters accounted for the largest proportion of the total volatiles content, and alcohols, aldehydes, ketones, and nitrogen heterocyclic compounds also accounted for relatively high proportions (Figure 2). The tea plants grown at different altitudes were ranked, from highest total aroma compound content to lowest, as follows: high altitude (26,227.13 μg/kg), low altitude (23,431.05 μg/kg), and then medium altitude (15,557.94 μg/kg) (Figure 2). Among the esters, those with the highest concentrations were *cis*-jasmine lactone (1974–8196 μg/kg) and δ-dodecalactone (2437–3410 μg/kg). Among the alcohols, those with relatively high concentrations were: hotrienol with a honey fragrance (1100–2664 μg/kg), linalool with a floral fragrance (421.8–701.3 μg/kg), and geraniol with a rose fragrance (733.4–910.7 μg/kg) [1,5]. The aldehyde butanal, 2-methyl- was present at relatively high concentrations (803.4–1441 μg/kg), as was the ketone (*Z*)-jasmone (392.9–1139 μg/kg). Among the nitrogenous compounds, indole (873.4–3882 μg/kg) showed the highest concentration.

**Figure 3:**
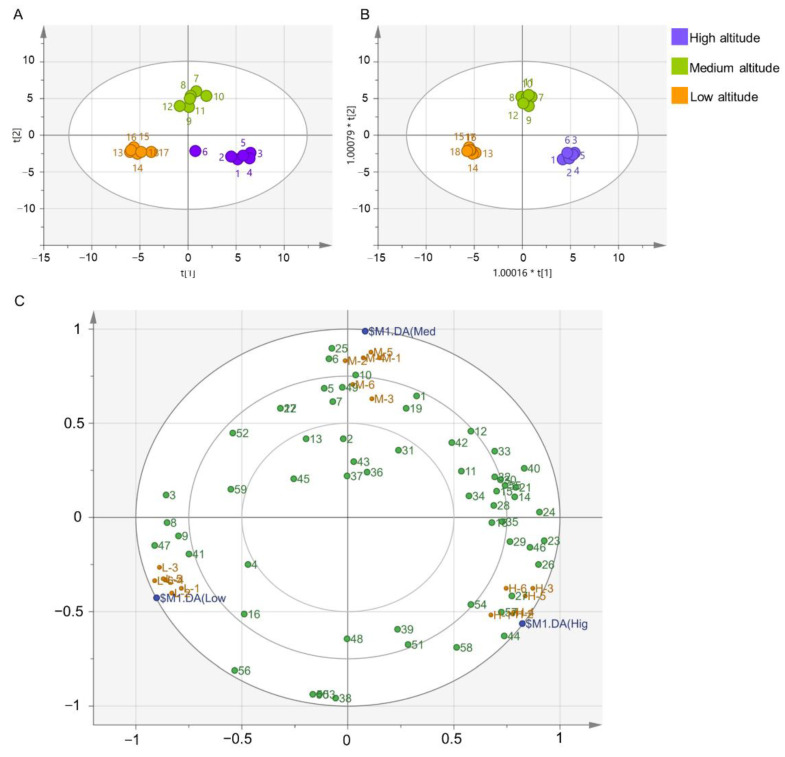
Multivariate chemometric analysis of Lingtou Dancong oolong tea product from different altitudes. (**A**) PCA score plot; (**B**) OPLS-DA score plot; (**C**) Loading plot. The horizontal axis represents PC1, and the vertical axis represents PC2. Hig, high altitude, Med, medium altitude, Low, low altitude. Compounds numbered in (**C**) were correspond to Table 1. High altitude, medium altitude and low altitude represent the Lingtou Dancong oolong teas from high, medium and low altitude, respectively.

Aroma components are important indicators for evaluating tea quality, and multivariate chemometric approaches are often used to analyze the diversity of sensory characteristics and chemical components of tea samples. Raw data were subjected to unsupervised PCA analysis and supervised OPLA-DA analysis (Figure 3). In the PCA, the first principal component (PC1, *X*-axis) explained 34.3% of the variation in total aroma, and the second principal component (PC2, *Y*-axis) explained 22.8%. The samples from three different altitudes formed different clusters on the plot. The OPLS-DA model had R^2^X of 0.784, R^2^Y of 0.99, Q^2^ of 0.963. Cross validation and permutation tests showed that the intercept between the blue regression line and the *Y* axis was less than 0, and all values of R^2^ and Q^2^ were lower than the original values, indicating that the model was not over-fitted and had good applicability and predictability (Figure S1). The compounds in the loading plots are referred to using serial numbers corresponding to the compounds in Table 1 and Figure 3C. The loading plot showed that hotrienol (23), nerol oxide (26), α-ionone (46), (*Z*)-jasmone (44) were present at higher levels in high-altitude tea than in medium- and low-altitude teas (Figure 3C). Compounds such as 5-ethyl-6-methyl-3*E*-hepten-2-one (25), (*Z*)-4-hepten (6), 1-octen-3-ol (10) were present at higher levels in the medium-altitude tea than in the high- and low-altitude teas. The contents of isoeugenol (47) and 1,2-dihydro-1,1,6-trimethyl naphthalene (41) were higher in low-altitude tea than in medium- and high-altitude teas. These compounds had relatively important effects on the aroma quality of tea made from plants grown at different altitudes.

### 3.2. GC-O/MS Analysis to Determine Contribution of Odorants to Aroma of Lingtou Dancong Oolong Tea Made from Plants Grown at Different Altitudes

**Figure 4:**
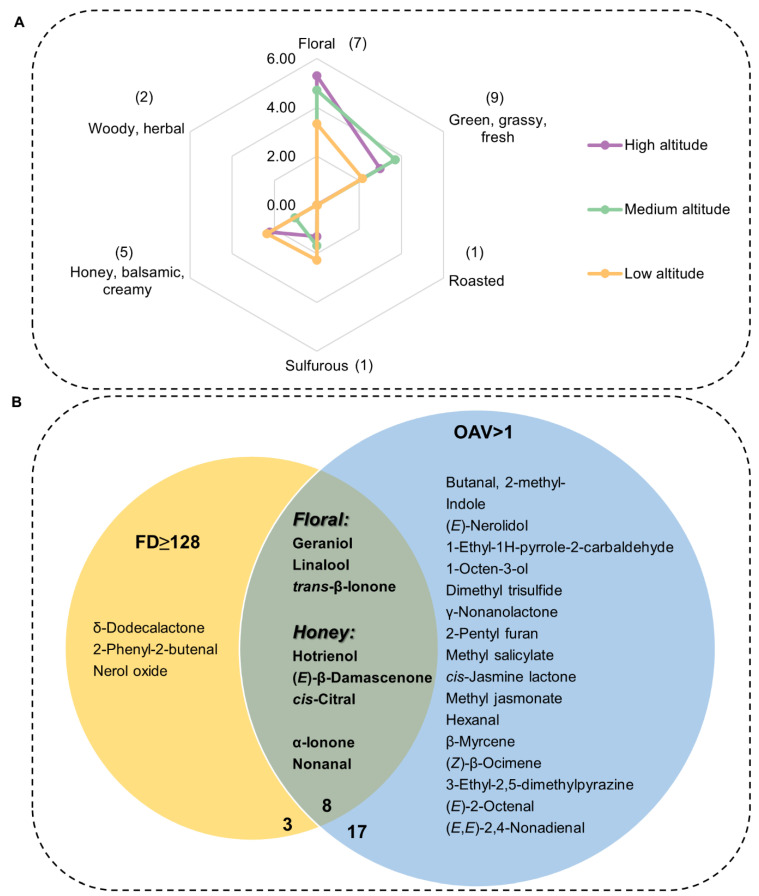
Aroma profile and key contributors of Lingtou Dancong oolong tea. (**A**) Specific scores were obtained by conversion according to OAV, and the numbers in brackets indicated the number of compounds; (**B**) Most important aroma contributors in Lingtou Dancong oolong tea identified according to FD and OAV. FD, flavor dilution factor; OAV, odor activity value. The number bellowed the circle indicates the number of compounds. High altitude, medium altitude and low altitude represent the Lingtou Dancong oolong teas from high, medium and low altitude, respectively.

The contribution of volatiles to the aroma profile not only depends on their content but also on their threshold. GC-O/MS analysis was performed to further identify key contributors to the overall aroma profile of the various tea materials. The results of GC-O/MS are shown in Table 2, and detailed descriptions of each odorant and its intensity are summarized in Tables S3–S5. Key aroma compounds were identified on the basis of their OAV, where a higher OAV indicates a major contribution to aroma [6]. The odor thresholds of compounds were obtained from the literature (Table S1). As shown in Table 2, 25 compounds had OAV > 1 at all altitudes. Among these, 12 were floral or honey odor compounds (Table 2). Linalool, with the highest OAVs (OAV = 1917.27–3187.73), contributed the major floral fragrance; (*E*, *E*)-2,4-nonadienal (OAV = 453.50–713.33) contributed the major green flavor; and (*E*)-β-damascenone (OAV = 316–357.00) and *cis*-jasmine lactone (OAV = 282.00–1170.86) contributed the major honey fragrance. Some unpleasant flavors such as the onion-flavored dimethyl trisulfide (OAV = 827.00–1438.00, sulfurous) also contributed to the overall aroma. All OAV values were converted by setting the highest OAV (3187.73) to 5, indicating the highest contribution. A radar map constructed using the converted OAV scores (Figure 4A) showed that the overall aroma profile of Lingtou Dancong oolong tea mainly consisted of floral, honey, and green odors. The floral fragrance of tea was richer in medium- and high-altitude teas and the honey fragrance was richer in high- and low-altitude teas. The green odor was more obvious, and the unpleasant onion odor was weaker in medium- and high-altitude teas than in low-altitude teas (Figure 4A).

Although OAV is a recognized method for evaluating aroma contribution, GC-O/MS results can be affected by the tea matrix, and odor perception is very complex and can be affected by multiple factors [11]. Therefore, it is important to analyze aroma contributions from multiple perspectives. The FD factor of aroma compounds was determined in order to clarify their contributions to the aroma profile of Lingtou Dancong oolong tea. All of the detected odor compounds had an FD factor of >16. The highest FD factor (512) indicated the greatest contribution, and the compounds with this FD value were floral or honey odor compounds: i.e., hotrienol (honey), *cis*-citral (honey), D-β-damascenone (honey), and *trans*-β-ionone (floral). Eleven aroma compounds had FD factors higher than 128 at all three altitudes: three were floral aroma compounds, four were honey aroma compounds, three were green aroma compounds, and one was a woody aroma compound (Table 2). These results were consistent with the organoleptic aroma profile (Figure 4).

### 3.3. Contents of Linalool and Its Precursor GPP in Fresh Leaves from Tea Plants Grown at Different Altitudes

**Figure 5:**
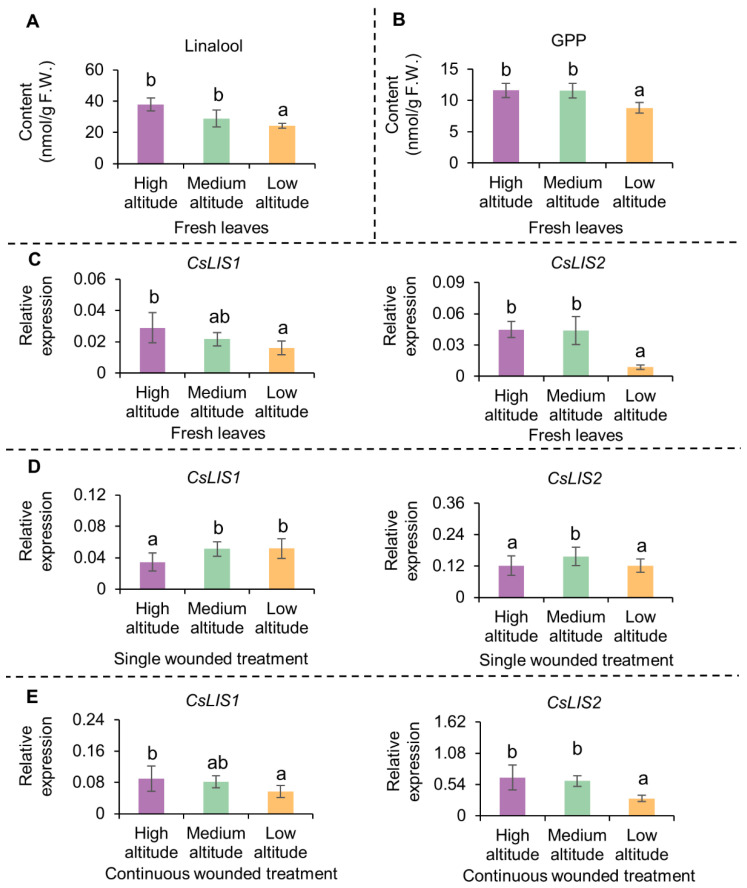
Contents of linalool, GPP and expression level of *CsLISs* in fresh leaves and wounded leaves of Lingtou Dancong grown at different altitudes. (**A**) Contents of linalool in fresh leaves; (**B**) Contents of GPP in fresh leaves. GPP, geranyl diphosphate; (**C**) Expression levels of *CsLIS1* and *CsLIS2* in fresh leaves (*CsEF-1α* as reference gene). *LIS*, *linalool synthase*.; (**D**) Expression levels of *CsLIS1* and *CsLIS2* in leaves after single wounded treatment for 3 h (*CsEF-1α* as reference gene); (**E**) Expression levels of *CsLIS1* and *CsLIS2* in leaves after continuous wounded treatment for 3 h (*CsEF-1α* as reference gene). The contents of GPP were determined indirectly based on the peak area of linalool from GPP hydrolysis. F.W. represents fresh weight. Data are expressed as mean ± S. D. (n = 3). Means distinguished with different letters are significantly different from each other among high altitude, medium altitude and low altitude (*p* ≤ 0.05). High altitude, medium altitude and low altitude represents the *C. sinensis* cv. Lingtou Dancong leaves picked from high, medium and low altitude, respectively.

The contents of linalool and geraniol were higher in high- and medium-altitude teas than in low-altitude tea. The OAVs of linalool and geraniol were higher than 1 and their FD values were higher than 128, indicating that they were major contributors to the floral aroma. Linalool and geraniol are both generated by the terpene metabolic pathway and share a common precursor, GPP. To explore the reasons for the higher contents of linalool and geraniol in tea plants grown at high and medium altitudes, we determined the contents of linalool and GPP in fresh leaves. The results showed that the contents of both linalool and GPP were higher in fresh leaves from plants grown at high and medium altitudes than in those from plants grown at a low altitude (Figure 5A,B).

The transcript levels of the *CsLISs* encoding linalool synthases in fresh leaves and leaves after wounding treatment were also analyzed (Figure 2 and Figure S2). The transcript levels of *CsLISs* were higher in fresh leaves of plants grown at medium and high altitudes than in those of plants grown at a low altitude, and were increased by the continuous wounding treatment. After the 3 h continuous wounding treatment, the transcript levels of *CsLISs* were higher in fresh leaves from plants grown at medium and high altitudes than in fresh leaves of those grown at low altitudes.

## 4. Discussion

### 4.1. Differences in Aroma Composition of Lingtou Dancong Oolong Tea among Plants Grown at Different Altitudes

In the evaluation of oolong tea, sensory evaluation is widely used to score the aroma quality. However, this method is subjective. The combination of GC-MS with an organoleptic analysis can evaluate the aroma of oolong tea more objectively. The GC-O/MS system is useful for identifying the contribution of aroma-active compounds in a complex matrix. We found that the categories of odorants in Lingtou Dancong oolong tea products from different altitudes were the same, but the content and proportion of each category differed among materials from different altitudes. The contents of esters (mainly *cis*-jasmine lactone) and nitrogen heterocyclic compounds (mainly indole) were lower in medium-altitude teas than in high- or low-altitude teas (Figure 2 and Table 1), resulting in the lowest total aroma compound content in medium-altitude tea. As a result of *cis*-jasmine lactone’s (honey) low odor threshold, the low content of *cis*-jasmine lactone in the medium-altitude tea also directly led to the deficiency of a honey fragrance (Table 1 and Table 2, Figure 4). Comparing tea materials from all altitudes, the total proportions of alcohols, aldehydes, terpenes, and nitrogen heterocyclic compounds tended to first increase, and then decrease with elevation (Figure 2). Another study found that aldehydes accounted for the largest proportion of odor compounds in most oolong teas [5]. However, we found that esters accounted for the largest proportion in Lingtou Dancong oolong tea. For example, the contents of γ-nonanolactone and γ-octanolactone, which confer the coconut fragrance of green tea, were significantly higher in medium- and high-altitude teas than in the low-altitude tea [12,13]. Among the alcohols, linalool and geraniol share the same precursor, GPP, but they have different odors [14]. Linalool has a floral-orange fragrance, while geraniol has an obvious rose fragrance [15]. Among the tea products, those from high and medium altitudes had higher linalool and geraniol contents in the fresh leaves, and higher contents of their shared precursor GPP (Table 1 and Figure 5). Aldehydes in tea can be produced through lipid oxidation and Strecker degradation under thermal conditions [16]. For example, hexanal (OAV > 1) and (*E*, *E*)-2,4-nonadienal (OAV > 1) are generated by lipid oxidation, and butanal, 2-methyl- (OAV > 1) is produced by Strecker degradation. We found that the contents of all three of these compounds were higher in medium- and high-altitude teas than in low-altitude tea, and they were identified as the main contributors of the green grass odor in tea. Among the ketones, α-ionone (OAV > 1), which has a woody fragrance, was present at higher levels in high-altitude tea than in medium- and low-altitude teas. (*E*)-β-damascenone (OAV > 1) and *trans*-β-ionone (OAV > 1) are two representative odor compounds generated by carotenoid degradation, and both are essential contributors to aroma [5,17]. As reported in previous studies, indole is another important aroma substance in oolong tea. The accumulation of indole is caused by turning-over during oolong tea processing, and this contributes to the floral fragrance [18].

We used OAV and FD analyses to evaluate whether a single odor compound makes a major contribution to fragrance. We detected eight odor compounds with FD ≥ 128 and OAV > 1 as the highest contributors, and they also had high sensory intensity (Figure 4B, Tables S3–S5). Among the eight fragrances, the contents of hotrienol (honey), linalool (floral), geraniol (floral), and α-ionone (woody, herbal) were positively correlated with altitude, while the *trans*-β-ionone (floral) content was highest in medium-altitude tea (Table 2). The *cis*-citral (honey) content was significantly higher in medium-and high-altitude teas than in low-altitude tea. Nonanal (green), which is a malt aroma, was present at significantly higher levels in medium-altitude teas than in low- or high-altitude teas [5]. The olfactory characteristics of these eight odor compounds were consistent with the stronger floral fragrance of high- and medium-altitude teas than low-altitude teas (Figure 4A). Hotrienol (honey), linalool (floral), and geraniol (floral) share the same terpene metabolic pathway. Altitude is negatively correlated with temperature, and cold stress can induce the release of linalool and geraniol [19]. Thus, environmental factors associated with altitude might affect the metabolism of aroma compounds in the terpene metabolic pathway, thereby affecting the aroma components and fragrance of tea. Overall, the honey and floral fragrances were prominent in high-altitude tea, and the green odor was weaker, meaning high-altitude tea had the best aroma quality.

Although all of the above odorants were screened based on the criteria of OAV > 1, there are many reasons why the OAV of a compound may be less than 1, such as a higher threshold or lower content of that compound. In addition, the tea matrix can affect the release of odorants [6]. Masking, synergistic, and additive effects among odorants can also affect perception [20]. Therefore, odorants with OAV < 1 can still contribute to overall aroma, but further research is required to explore their contributions and interactions in more detail.

### 4.2. Aroma Characteristics of Lingtou Dancong Oolong Tea

Lingtou Dancong, also known as Baiye Dancong, like Fenghuang Dancong, is a protected geographical indication product (PGI) in Guangdong province, China. The aroma of oolong tea from Guangdong province differs from those of other oolong teas because of the varieties used and the processing methods. Tea tasting experience and existing literature indicate that the floral and honey fragrances are key sensory characteristics of Dancong oolong tea [2,21]. Other Dancong oolong teas have a prominent floral fragrance, while Lingtou Dancong has a unique honey fragrance [2,21]. The literature indicates that linalool and linalool oxides are important substances for forming the honey fragrance [2,22]. However, linalool is a vital odor compound, not only in Lingtou Dancong oolong tea, but also in other oolong teas, green tea, black tea, and even dark tea with a higher degree of fermentation [7,23,24,25]. Therefore, linalool and its oxides are important and universal substances in tea, rather than the signature aroma of Lingtou Dancong oolong tea. The oolong tea “Oriental beauty” is widely known for its honey fragrance, which is largely conferred by hotrienol [26]. Hotrienol, which is present in honey and has a honey and fruit aroma, is a very unstable thermal product that can be converted from diols or linalool [27,28]. Studies have shown that the hotrienol content is higher in Lingtou Dancong oolong tea than in other Dancong oolong teas, and that hotrienol is not present in Rougui teas with different degrees of baking and fermentation [2,29,30]. In our study, we detected higher contents of both hotrienol and its precursor, linalool, in high- and medium-altitude teas than in low-altitude tea. This result suggests that hotrienol in tea might be converted from linalool, whose content might be related to altitude. (*E*)-β-Damascenone (OAV = 316–357) and *cis*-citral (OAV = 4.17–8.04) were also identified as key honey odorants with high OAVs, high sensory intensity (Tables S3–S5), and the highest FD (>512) among all odor compounds (Table 2). Notably, (*E*)-β-damascenone was not detected in any of the tea products made from Shuixian, Huangmeigui, and Zimudan (three varieties suitable for making oolong tea) [23]. *cis*-Citral was not detected in Dahongpao, Tieguanyin and Dongdingwulong teas [31]. Therefore, hotrienol, (*E*)-β-damascenone, and *cis*-citral may be the key components of the special honey aroma of Lingtou Dancong oolong tea. However, further aroma recombination experiments are required to test this hypothesis.

## 5. Conclusions

**Figure 6:**
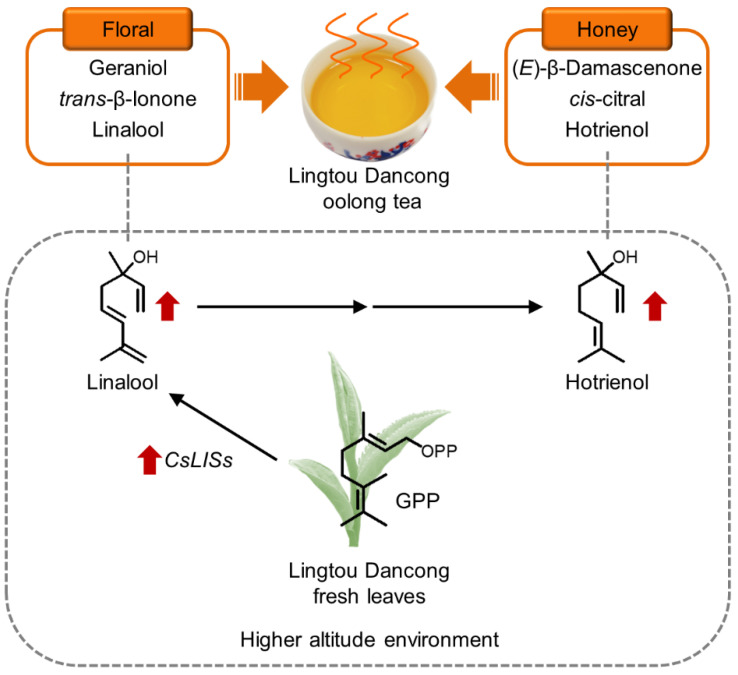
Aroma characteristics of Lingtou Dancong oolong tea and the effect of altitude on it. GPP, geranyl diphosphate; *LIS*, *linalool synthase*.

In this study, the SBSE method and GC-O/MS technique were used to evaluate the aroma components of Lingtou Dancong oolong tea made from plants grown at different altitudes. A total of 59 common aroma compounds were identified and quantified. The PCA and OPLS-DA confirmed differences in the aroma quality of tea from different altitudes. The categories of aroma compounds in teas from different altitudes were the same, but the contents and proportions were different. These differences, together with the thresholds of each aroma compound, affected the final aroma profile of the various teas. Using OAV (the information of threshold was referred to the literatures [5,6,12,31,32,33,34,35,36,37,38,39,40,41]) and FD analyses, the eight odor compounds making the largest contributions to aroma were screened, and most of them had honey and floral fragrances. Among them, hotrienol, (*E*)-β-damascenone, and *cis*-citral were the most noteworthy contributors to aroma (Figure 4B). All of them have a honey fragrance, and together they may confer the honey fragrance of Lingtou Dancong oolong tea. Their presence and contents may be related to the variety and/or the subsequent processing methods, but further analyses are required to explore these ideas in more detail. The contents of linalool (a key component of floral fragrance) and hotrienol (a key component of honey fragrance) varied with altitude. Its higher content at higher altitude was consistent with higher contents of its precursor, GPP, as well as higher transcript levels of *CsLISs* in the fresh leaves (Figure 6). The results of this study shed light on the aroma compounds that contribute to the fragrance of Lingtou Dancong oolong tea. Our results will be useful for devising strategies to control the contents of various aroma compounds in tea during cultivation and processing.

## Figures and Tables

**Table 1:** Qualitative and quantitative analysis of aroma of Lingtou Dancong tea product from plants grown at different altitudes.

NO	Odorants	RI ^a^ (CAL)	RI ^b^ (NIST)	Concentration (μg/kg, D.W.) ^c^			Category	Identification Method ^d^
				High	Medium	Low		
1	Butanal, 2-methyl-	704	662	1052 ± 302.9 a	1441 ± 339.2 b	803.4 ± 160.0 a	Aldehydes	RI, MS, O, STD
2	Hexanal	790	800	470.5 ± 59.74 a	624.9 ± 114.1 a	284.1 ± 20.73 a	Aldehydes	RI, MS, O, STD
3	Ethyl 2-methyl butyrate	838	849	14.64 ± 2.25 a	20.60 ± 2.94 b	25.18 ± 2.38 c	Esters	RI, MS, O, (*E*)-Methyl geranate (1189-09-9)
4	2-Heptanone	884	891	34.11 ± 6.06 a	47.67 ± 10.25 a	40.13 ± 2.80 a	Ketones	RI, MS, O, 2-Undecanone (112-12-9)
5	Heptanal	894	901	6.93 ± 1.52 a	51.19 ± 16.83 b	18.94 ± 2.68 a	Aldehydes	RI, MS, O, STD
6	(*Z*)-4-Heptenal	894	901	7.23 ± 0.98 a	29.16 ± 6.56 b	10.42 ± 1.15 a	Aldehydes	RI, MS, O, (*E*)-2-Nonenal (18829-56-6)
7	2,5-Dimethyl pyrazine	913	921	338.6 ± 41.19 a	402.0 ± 47.95 b	353.5 ± 37.25 ab	Nitrogen heterocyclic	RI, MS, O, Pyrazine, 2,6-diethyl- (13067-27-1)
8	Diethyl disulfide	914	927	14.66 ± 3.14 a	18.12 ± 3.46 b	25.04 ± 1.33 c	Sulfur-containing	RI, MS, O, Dimethyl trisulfide (3658-80-8)
9	Dimethyl trisulfide	967	970	8.27 ± 1.53 a	10.64 ± 1.38 a	14.38 ± 0.83 b	Sulfur-containing	RI, MS, O, STD
10	1-Octen-3-ol	976	980	12.26 ± 2.70 a	24.27 ± 2.65 b	12.60 ± 1.01 a	Alcohols	RI, MS, O, STD
11	Methyl heptenone	981	986	57.54 ± 10.18 b	57.66 ± 8.32 b	45.15 ± 3.79 a	Ketones	RI, MS, O, STD
12	β-Myrcene	985	991	117.57 ± 17.65 b	128.0 ± 22.72 b	86.17 ± 6.00 a	Terpenes	RI, MS, O, STD
13	2-Pentyl furan	986	993	62.04 ± 29.74 a	122.4 ± 60.65 a	89.84 ± 41.92 a	Others	RI, MS, O, STD
14	α-Phellandrene	1000	1005	22.90 ± 4.75 b	19.68 ± 3.02 b	12.82 ± 1.36 a	Terpenes	RI, MS, O,γ-Terpinene (99-85-4)
15	(*Z*)-β-Ocimene	1043	1038	100.13 ± 18.53 b	91.37 ± 13.79 b	65.66 ± 12.30 a	Terpenes	RI, MS, O, STD
16	1-Ethyl-1*H*-pyrrole-2-carbaldehyde	1048	1046	165.48 ± 40.37 a	143.67 ± 19.11 a	205.60 ± 21.76 b	Nitrogen heterocyclic	RI, MS, O, Pyrazine, 2,6-diethyl- (13067-27-1)
17	(*E*)-2-Octenal	1055	1060	26.07 ± 5.06 a	48.58 ± 20.03 b	36.83 ± 9.49 ab	Aldehydes	RI, MS, O, (*E*)-2-Nonenal (18829-56-6)
18	γ-Terpinene	1055	1060	17.99 ± 3.28 b	15.14 ± 2.40 ab	12.80 ± 0.61 a	Terpenes	RI, MS, O, STD
19	3-Ethyl-2,5-dimethylpyrazine	1076	1082	15.36 ± 2.12 ab	18.37 ± 2.39 b	13.44 ± 1.67 a	Nitrogen heterocyclic	RI, MS, O, Pyrazine, 2,6-diethyl- (13067-27-1)
20	Pyrazine, 2,6-diethyl-	1085	1084	trace	trace	trace	Nitrogen heterocyclic	RI, MS, O, STD
21	Linalool	1097	1099	701.3 ± 118.4 b	636.8 ± 81.2 b	421.8 ± 41.83 a	Alcohols	RI, MS, O, STD, *m*/*z* = 121
22	Nonanal	1100	1104	128.6 ± 26.82 a	210.1 ± 35.17 c	173.6 ± 25.65 b	Aldehydes	RI, MS, O, STD
23	Hotrienol	1109	1107	2664 ± 357.6 c	1825 ± 188.7 b	1100 ± 85.6 a	Alcohols	RI, MS, O, Linalool (78-70-6)
24	2,6-Dimethyl-1,3,5,7-octatetraene, *E*, *E-*	1129	1131	145.5 ± 25.26 a	110.1 ± 16.83 b	60.21 ± 4.88 c	Terpenes	RI, MS, O, (*Z*)-β-Ocimene (3338-55-4)
25	5-Ethyl-6-methyl-3*E*-hepten-2-one	1143	1144	43.59 ± 5.86 a	82.26 ± 11.41 b	50.49 ± 3.46 a	Ketones	RI, MS, O, Methyl heptenone (110-93-0)
26	Nerol oxide	1152	1153	251.0 ± 39.29 c	154.1 ± 19.87 b	104.4 ± 8.22 a	Oxides	RI, MS, O, *trans*-Linalool oxide (pyranoid) (39028-58-5)
27	*trans*-Linalool oxide (pyranoid)	1174	1173	217.18 ± 41.10 b	119.6 ± 23.09 a	106.2 ± 29.70 a	Oxides	RI, MS, O, STD
28	Terpinen-4-ol	1177	1177	11.52 ± 1.94 b	9.45 ± 2.11 ab	5.96 ± 0.52 a	Alcohols	RI, MS, O, STD
29	α-Terpineol	1192	1189	78.87 ± 11.95 a	63.14 ± 8.69 b	50.19 ± 6.84 c	Alcohols	RI, MS, O, Terpinen-4-ol (562-74-3)
30	Methyl salicylate	1195	1192	137.1 ± 22.00 b	129.7 ± 17.04 b	95.20 ± 8.14 a	Esters	RI, MS, O, STD
31	(*E*,*E*)-2,4-Nonadienal	1212	1216	42.26 ± 8.54 a	42.80 ± 7.81 a	27.21 ± 5.36 a	Aldehydes	RI, MS, O, (*E*)-2-Nonenal (18829-56-6)
32	Nerol	1227	1228	13.84 ± 2.29 b	12.79 ± 2.60 b	6.92 ± 1.13 a	Alcohols	RI, MS, O, Geraniol (106-24-1)
33	*cis*-Citral	1238	1240	39.18 ± 6.11 b	40.22 ± 5.13 b	20.86 ± 1.55 a	Aldehydes	RI, MS, O, STD
34	Geraniol	1253	1255	910.7 ± 139.5 b	868.4 ± 119.7 ab	733.4 ± 66.2 a	Alcohols	RI, MS, O, STD
35	γ-Octanolactone	1259	1261	22.49 ± 3.38 b	18.26 ± 4.04 ab	13.45 ± 1.18 a	Esters	RI, MS, O, γ-Nonanolactone (104-61-0)
36	2-Phenyl-2-butenal	1273	1279	78.45 ± 11.85 a	83.28 ± 11.48 a	76.50 ± 6.85 a	Aldehydes	RI, MS, O, α-Hexyl cinnamaldehyde (101-86-0)
37	2,4-Decadienal	1290	1295	102.58 ± 27.29 a	103.44 ± 18.68 a	79.33 ± 8.94 a	Aldehydes	RI, MS, O, (*E*)-2-Nonenal (18829-56-6)
38	Indole	1303	1295	3882 ± 687.2 b	873.4 ± 164.6 a	3814 ± 320.5 b	Nitrogen heterocyclic compounds	RI, MS, O, STD
39	Theaspirane B	1315	1311	7.73 ± 1.22 b	5.73 ± 0.82 a	6.84 ± 0.60 ab	Others	RI, MS, O, STD
40	(*E*)-Methyl geranate	1319	1324	15.82 ± 2.70 b	14.56 ± 1.94 b	7.30 ± 0.69 a	Ketones	RI, MS, O, STD
41	1,2-Dihydro-1,1,6-trimethylnaphthalene	1353	1354	173.6 ± 31.81 a	186.6 ± 30.53 a	244.4 ± 22.43 b	Others	RI, MS, O, α-Cadinol (481-34-5)
42	γ-Nonanolactone	1363	1363	91.50 ± 12.62 b	98.08 ± 18.98 b	72.04 ± 6.23 a	Ketones	RI, MS, O, STD
43	(*E*)-β-Damascenone	1382	1386	15.80 ± 2.21 a	17.87 ± 1.71 a	15.80 ± 1.71 a	Ketones	RI, MS, O, α-Ionone (127-41-3)
44	(*Z*)-Jasmone	1400	1394	1139.3 ± 97.84 b	392.9 ± 29.20 a	476.0 ± 29.20 a	Ketones	RI, MS, O, STD
45	Dehydrodihydroionone	1418	1424	11.95 ± 2.21 a	14.96 ± 2.21 a	14.77 ± 2.21 a	Ketones	RI, MS, O, α-Ionone (127-41-3)
46	α-Ionone	1425	1426	151.12 ± 22.22 c	110.7 ± 13.49 b	85.77 ± 6.86 a	Ketones	RI, MS, O, STD
47	Isoeugenol	1454	1454	155.5 ± 27.03 a	218.3 ± 34.96 b	382.3 ± 54.81 c	Others	RI, MS, O, (*Z*)-Jasmone (488-10-8)
48	γ-Decanolactone	1468	1470	14.85 ± 2.11 b	9.36 ± 1.73 a	14.44 ± 1.69 b	Ketones	RI, MS, O, STD
49	*trans*-β-Ionone	1490	1486	221.6 ± 31.15 a	288.1 ± 33.53 b	230.1 ± 9.51 a	Ketones	RI, MS, O, STD
50	*cis*-Jasmine lactone	1496	1518	7539 ± 501.6 b	1974 ± 165.5 a	8196 ± 450.5 b	Esters	RI, MS, O, STD
51	δ-Dodecalactone	1498	1496	3410 ± 248.9 b	2437 ± 200.4 a	2952 ± 156.7 ab	Esters	RI, MS, O, STD
52	Dihydroactinidioide	1538	1538	455.6 ± 52.40 a	813.8 ± 104.62 b	782.0 ± 88.58 b	Esters	RI, MS, O, STD
53	(*E*)-Nerolidol	1563	1564	587.0 ± 97.27 b	160.8 ± 22.32 a	620.8 ± 21.22 b	Alcohols	RI, MS, O, Geraniol (106-24-1)
54	Hexyl benzoate	1576	1580	2.15 ± 0.44 b	0.46 ± 0.08 a	0.02 ± 0.00 a	Esters	RI, MS, O, Benzyl Benzoate (120-51-4)
55	Caryophyllene oxide	1593	1581	2.17 ± 0.40 b	2.08 ± 0.24 b	1.84 ± 0.19 a	Oxides	RI, MS, O, Theaspirane B (36431-72-8)
56	Methyl jasmonate	1646	1638	83.84 ± 10.05 b	26.00 ± 3.96 a	137.03 ± 12.05 c	Esters	RI, MS, O, STD
57	α-Cadinol	1657	1653	76.23 ± 11.72 a	49.33 ± 5.71 b	49.08 ± 5.11 b	Alcohols	RI, MS, O,δ-Cadinene (483-76-1)
58	Bisabolol oxide B	1656	1655	55.59 ± 6.06 b	36.51 ± 4.73 a	42.75 ± 3.45 a	Oxides	RI, MS, O, *trans*-Linalool oxide (furanoid) (34995-77-2)
59	*cis*-3-Hexenyl salicylate	1681	1670	2.64 ± 0.60 a	3.54 ± 0.74 ab	4.05 ± 0.29 b	Esters	RI, MS, O, Methyl salicylate (119-36-8)

^a^: retention index on a HP-5MS column, the retention index is calculated from the standard (C8–C40). ^b^: retention index according to NIST14 database (2014). ^c^: D.W., dry weight. Data are expressed as mean ± S. D. (*n* = 6). Means distinguished with different letters are significantly different from each other among high altitude, medium altitude and low altitude (*p* ≤ 0.05). ^d^: RI, identified by retention index, the retention index is calculated from the standard (C8–C40) and compared with NIST14 database; MS, identified by mass spectrum comparisons; O, identified by sniffing; STD, confirmed by authentic standards. Qualitative analysis was performed by a combination of NIST14 database comparisons, RI values and olfactive evaluation. Accurate quantification was performed by authentic standards. Some standards were not available, so those with similar structures were selected as standards for quantitative analysis. During the extraction, 1 g tea powder was brewed in 10 mL water (100 °C) for GC-O/MS analysis. High, medium and low represents the Lingtou Dancong oolong tea from high, medium and low altitude, respectively.

**Table 2:** Analysis of aroma contribution of Lingtou Dancong Oolong tea product from plants grown at different altitudes.

NO	Odorants	OT ^a^ (μg/kg)		OAV ^b^		FD ^c^			Olfactive Family
			High	Medium	Low	High	Medium	Low	
1	Butanal, 2-methyl-	1	1052.00	1441.00	803.42	>64	>64	>64	Green, grassy, fresh
2	Hexanal	4.5	104.44	138.87	63.13	>32	>32	>32	Green, grassy, fresh
3	Ethyl 2-methyl butyrate	n.f.	-	-	-	<16	<16	<16	Fruity
4	2-Heptanone	140	0.24	0.34	0.29	<16	<16	<16	Green, grassy, fresh
5	Heptanal	550	0.01	0.09	0.03	>64	>64	>32	Fatty, oil
6	(*Z*)-4-Heptenal	10	0.72	2.92	1.04	>16	>64	>32	Fatty, oil
7	2,5-Dimethyl pyrazine	n.f.	-	-	-	>16	>32	>32	Roasted
8	Diethyl disulfide	30	0.49	0.60	0.83	>32	>32	>32	Sulfurous
9	Dimethyl trisulfide	0.01	827.00	1064.00	1438.00	>16	>16	>32	Sulfurous
10	1-Octen-3-ol	1.5	8.17	16.18	8.40	>64	>128	>64	Green, grassy, fresh
11	Methyl heptenone	100	0.58	0.58	0.45	>64	>64	>32	Green, grassy, fresh
12	β-Myrcene	14	8.40	9.14	6.16	>64	>128	>64	Green, grassy, fresh
13	2-Pentyl furan	5.9	10.52	20.75	15.23	>64	>256	>128	Green, grassy, fresh
14	α-Phellandrene	n.f.	-	-	-	>32	>32	<16	Green, grassy, fresh
15	(*Z*)-β-Ocimene	34	2.95	2.69	1.93	>32	>16	>16	Floral
16	1-Ethyl-1*H*-pyrrole-2-carbaldehyde	37	4.47	3.88	5.56	>32	>32	>32	Woody, herbal
17	(*E*)-2-Octenal	3	8.69	16.19	12.28	>32	>32	>32	Green, grassy, fresh
18	γ-Terpinene	55	0.33	0.28	0.23	>32	<16	<16	Woody, herbal
19	3-Ethyl-2,5-dimethylpyrazine	8.6	1.79	2.14	1.56	>32	>64	>16	Roasted
20	Pyrazine, 2,6-diethyl-	n.f.	-	-	-	>128	>128	>64	Roasted
21	Linalool	0.22	3187.73	2894.55	1917.27	>256	>256	>128	Floral
22	Nonanal	40	3.22	5.25	4.34	>128	>128	>128	Green, grassy, fresh
23	Hotrienol	110	24.22	16.59	10.00	>512	>512	>512	Honey, balsamic, creamy
24	2,6-Dimethyl-1,3,5,7-octatetraene, *E*, *E*-	n.f.	-	-	-	>32	>32	>32	Woody, herbal
25	5-Ethyl-6-methyl-3*E*-hepten-2-one	n.f.	-	-	-	>64	>64	>32	Green, grassy, fresh
26	Nerol oxide	n.f.	-	-	-	>256	>256	>128	Green, grassy, fresh
27	*trans*-Linalool oxide (pyranoid)	320	0.68	0.37	0.33	>32	>16	>16	Woody, herbal
28	Terpinen-4-ol	4370	0.00	0.00	0.00	>32	>32	>32	Woody, herbal
29	α-Terpineol	330	0.24	0.19	0.15	>64	>64	>32	Woody, herbal
30	Methyl salicylate	40	3.43	3.24	2.38	>32	>32	>16	Green, grassy, fresh
31	(*E*, *E*)-2,4-Nonadienal	0.06	704.33	713.33	453.50	>64	>64	>64	Green, grassy, fresh
32	Nerol	300	0.05	0.04	0.02	>16	>16	>16	Green, grassy, fresh
33	*cis*-Citral	30	7.84	8.04	4.17	>512	>512	>512	Honey, balsamic, creamy
34	Geraniol	40	22.77	21.71	18.34	>256	>128	>128	Floral
35	γ-Octanolactone	n.f.	-	-	-	>128	>64	>32	Honey, balsamic, creamy
36	2-Phenyl-2-butenal	500	0.16	0.17	0.15	>128	>128	>128	Green, grassy, fresh
37	2,4-Decadienal	n.f.	-	-	-	>32	>32	>16	Honey, balsamic, creamy
38	Indole	100	38.82	8.73	38.14	>128	>64	>128	Floral
39	Theaspirane B	n.f.	-	-	-	>32	>32	>32	Woody, herbal
40	(*E*)-Methyl geranate	n.f.	-	-	-	>64	>64	>64	Woody, herbal
41	1,2-Dihydro-1,1,6-trimethylnaphthalene	n.f.	-	-	-	>32	>32	>64	Floral
42	γ-Nonanolactone	27	7.25	7.77	5.71	>64	>128	>64	Honey, balsamic, creamy
43	(*E*)-β-Damascenone	0.05	316.00	357.40	316.00	>512	>512	>512	Honey, balsamic, creamy
44	(*Z*)-Jasmone	21,600	0.05	0.02	0.02	>64	>32	>32	Floral
45	Dehydrodihydroionone	n.f.	-	-	-	>32	>32	>32	Floral
46	α-Ionone	76	1.99	1.46	1.13	>256	>256	>256	Woody, herbal
47	Isoeugenol	n.f.	-	-	-	>32	>32	>64	Floral
48	γ-Decanolactone	n.f.	-	-	-	>128	>64	>128	Honey, balsamic, creamy
49	*trans*-β-Ionone	7	31.66	41.16	32.87	>512	>512	>512	Floral
50	*cis*-Jasmine lactone	7	1077.00	282.00	1170.86	>128	>64	>128	Honey, balsamic, creamy
51	δ-Dodecalactone	n.f.	-	-	-	>128	>128	>128	Honey, balsamic, creamy
52	Dihydroactinidioide	500	0.91	1.63	1.56	>16	>16	>16	Woody, herbal
53	(*E*)-Nerolidol	10	58.70	16.08	62.08	>64	>32	>32	Floral
54	Hexyl benzoate	73	0.03	0.01	0.00	>16	<16	<16	Green, grassy, fresh
55	Caryophyllene oxide	n.f.	-	-	-	<16	<16	<16	Floral
56	Methyl jasmonate	3	27.95	8.67	45.68	>16	<16	>32	Floral
57	α-Cadinol	n.f.	-	-	-	>64	>32	>32	Woody, herbal
58	Bisabolol oxide B	n.f.	-	-	-	>64	>32	>32	Woody, herbal
59	*cis*-3-Hexenyl salicylate	115	0.01	0.03	0.04	>32	>64	>64	Floral

^a^: Odor thresholds in water. The values were according to the literatures. n.f., data was not found in the literature. Detailed literature is listed in Table S1. ^b^: Odor activity value, ratio of odorant concentration in water. The calculation of OAV is based on the average value of aroma contents and OTs. ^c^: Flavor dilution factor. During the extraction, 1 g tea powder was brewed in 10 mL water (100 °C) for GC-O/MS analysis. High, medium and low represent the Lingtou Dancong oolong teas from high, medium and low altitude, respectively.

## Data Availability

The data presented in this study are available within the article and Supplementary Materials.

## Supplementary Materials

The following supporting information can be downloaded at: https://www.mdpi.com/article/10.3390/metabo12111063/s1, Figure S1: OPLS-DA model validation; Figure S2: Expression level of *CsLISs* in fresh leaves and wounded leaves of Lingtou Dancong grown at different altitudes; Table S1: Threshold information; Table S2: The primers used for quantitative real time PCR (qRT-PCR) in the study; Table S3: Detailed information of Lingtou Dancong tea product at high altitude; Table S4: Detailed information of Lingtou Dancong tea product at medium altitude; Table S5: Detailed information of Lingtou Dancong tea product at low altitude.

## References

[1] Zeng L.T., Zhou X.C., Su X.G., Yang Z.Y. (2020). Chinese oolong tea: An aromatic beverage produced under multiple stresses. Trends Food Sci. Tech..

[2] Chen W., Hu D., Miao A.Q., Qiu G.J., Qiao X.Y., Xia H.L., Ma C.Y. (2022). Understanding the aroma diversity of Dancong tea (*Camellia sinensis*) from the floral and honey odors: Relationship between volatile compounds and sensory characteristics by chemometrics. Food Control.

[3] Zhou C.J., Zhuang D.H., Guo S.J., Zhu H., Ma R.J., Wu Q.H. (2014). Classification and identification of different aromatics in tea made from different cultivar of Fenghuang dancing. J. Tea Sci..

[4] Tang H., Tang J.C., Cao J.X., Zhou B., Li J.L., Cai J. (2015). Analysis of quality differences among Fenghuang dancong tea in different altitude ranges. Chin. Agric. Sci. Bull..

[5] Zhai X.T., Zhang L., Granvogl M., Ho Q.T., Wan X.C. (2022). Flavor of tea (*Camellia sinensis*): A review on odorants and analytical techniques. Compr. Rev. Food Sci. Food Saf..

[6] Wang M.Q., Ma W.J., Shi J., Zhu Y., Lin Z., Lv H.P. (2020). Characterization of the key aroma compounds in Longjing tea using stir bar sorptive extraction (SBSE) combined with gas chromatography-mass spectrometry (GC-MS), gas chromatography-olfactometry (GC-O), odor activity value (OAV), and aroma recombination. Food Res. Int..

[7] Zhu Y., Yan H., Zhang Z.F., Zeng J.M., Zhang Y., Wang M.Q., Peng Q.H., Lv H.P., Lin Z. (2021). Assessment of the contribution of chiral odorants to aroma property of baked green teas using an efficient sequential stir bar sorptive extraction approach. Food Chem..

[8] Zhu Y., Shao C.Y., Lv H.P., Zhang Y., Dai W.D., Guo L., Tan J.F., Peng Q.H., Lin Z. (2017). Enantiomeric and quantitative analysis of volatile terpenoids in different teas (*Camellia sinensis*). J. Chromatogr. A.

[9] Lv H.P., Zhong Q.S., Lin Z., Wang L., Tan J.F., Guo L. (2012). Aroma characterisation of Pu-erh tea using headspace-solid phase microextraction combined with GC/MS and GC-olfactometry. Food Chem..

[10] Jian G.T., Jia Y.X., Li J.L., Zhou X.C., Liao Y.Y., Dai G.Y., Zhou Y., Tang J.C., Zeng L.T. (2021). Elucidation of the regular emission mechanism of volatile beta-ocimene with anti-insect function from tea plants (*Camellia sinensis*) exposed to herbivore attack. J. Agric. Food Chem..

[11] Chen X.H., Sun H.Y., Qu D., Yan F., Jin W.G., Jiang H., Chen C., Zhang Y.F., Li C.Y., Xu Z.M. (2020). Identification and characterization of key aroma compounds in Chinese high altitude and northernmost black tea (*Camellia sinensis*) using distillation extraction and sensory analysis methods. Flavour Frag. J..

[12] Flaig M., Qi S., Wei G.D., Yang X.G., Schieberle P. (2020). Characterization of the key odorants in a high-grade Chinese green tea beverage (*Camellia sinensis*; jingshan cha) by means of the sensomics approach and elucidation of odorant changes in tea leaves caused by the tea manufacturing process. J. Agric. Food Chem..

[13] Flaig M., Qi S.C., Wei G.D., Yang X.G., Schieberle P. (2020). Characterisation of the key aroma compounds in a Longjing green tea infusion (*Camellia sinensis*) by the sensomics approach and their quantitative changes during processing of the tea leaves. Eur. Food Res. Technol..

[14] Zeng L.T., Watanabe N., Yang Z.Y. (2019). Understanding the biosyntheses and stress response mechanisms of aroma compounds in tea (*Camellia sinensis*) to safely and effectively improve tea aroma. Crit. Rev. Food Sci..

[15] Burdock G.A. (2010). Fenarolis Handbook of Flavor Ingredients.

[16] Ho C.T., Zheng X., Li S.M. (2015). Tea aroma formation. Food Sci. Hum. Wellness.

[17] Lin Q., Ni H., Wu L., Weng S.Y., Li L.J., Chen F. (2021). Analysis of aroma-active volatiles in an SDE extract of white tea. Food Sci. Nutr..

[18] Zeng L.T., Zhou Y., Gui J.D., Fu X.M., Mei X., Zhen Y.P., Ye T.X., Du B., Dong F., Watanabe N. (2016). Formation of volatile tea constituent indole during the oolong tea manufacturing process. J. Agric. Food Chem..

[19] Zhao M.Y., Wang L., Wang J.M., Jin J.Y., Zhang N., Lei L., Gao T., Jing T.T., Zhang S.R., Wu Y. (2020). Induction of priming by cold stress via inducible volatile cues in neighboring tea plants. J. Integr. Plant Biol..

[20] Niu Y.W., Ma Y.W., Xiao Z.B., Zhu J.C., Xiong W., Chen F. (2022). Characterization of the key aroma compounds of three kinds of chinese representative black tea and elucidation of the perceptual interactions of methyl salicylate and floral odorants. Molecules.

[21] Zhao J., Liu W.W., Chen Y., Zhang X., Wang X., Wang F.H., Qian Y.Z., Qiu J. (2022). Identification of markers for tea authenticity assessment: Non-targeted metabolomics of highly similar oolong tea cultivars (*Camellia sinensis* var. sinensis). Food Control.

[22] Ma C.Y., Li J.X., Chen W., Wang W.W., Qi D.D., Pang S., Miao A.Q. (2018). Study of the aroma formation and transformation during the manufacturing process of oolong tea by solid-phase micro-extraction and gas chromatography-mass spectrometry combined with chemometrics. Food Res. Int..

[23] Guo X.Y., Schwab W., Ho C.T., Song C.K., Wan X.C. (2021). Characterization of the aroma profiles of oolong tea made from three tea cultivars by both GC-MS and GC-IMS. Food Chem..

[24] Liu H.C., Xu Y.J., Wen J., An K.J., Wu J.J., Yu Y.S., Zou B., Guo M.H. (2021). A comparative study of aromatic characterization of Yingde black tea infusions in different steeping temperatures. LWT-Food Sci. Technol..

[25] Wang C., Li J., Wu X.J., Zhang Y., He Z.G., Zhang Y., Zhang X.M., Li Q., Huang J.A., Liu Z.H. (2022). Pu-erh tea unique aroma: Volatile components, evaluation methods and metabolic mechanism of key odor-active compounds. Trends Food Sci. Tech..

[26] Ogura M., Terada I., Shirai F., Tokoro K., Chen K.R., Chen C.L., Lin M.L., Shimizu B., Kinoshita T., Sakata K. Tracing aroma characteristics changes during processing of the famous Formosa oolong tea “Oriental Beauty”. Proceedings of the 2004 International Conference on O-Cha (tea) Culture and Science.

[27] Jerkovic I., Kus P.M. (2014). Terpenes in honey: Occurrence, origin and their role as chemical biomarkers. RSC Adv..

[28] Tsitsishvili V., Ramishvili T., Ivanova I., Kokiashvili N., Bukia T., Kurtsikidze G. (2019). Linalool oxidation reaction with air under ultrasound and microwave irradiations. Bull. Georg. Natl. Acad. Sci..

[29] Yang P., Yu M.G., Song H.L., Xu Y.Q., Lin Y.P., Granvogl M. (2022). Characterization of key aroma-active compounds in Rough and moderate fire rougui wuyi rock tea (*Camellia sinensis*) by sensory-directed flavor analysis and elucidation of the influences of roasting on aroma. J. Agric. Food Chem..

[30] Liu Z.B., Chen F.C., Sun J.Y., Ni L. (2022). Dynamic changes of volatile and phenolic components during the whole manufacturing process of Wuyi Rock tea (Rougui). Food Chem..

[31] Zhu J.C., Chen F., Wang L.Y., Niu Y.W., Yu D., Shu C., Chen X.H. (2015). Comparison of aroma-active volatiles in oolong tea infusions using GC-Olfactometry, GC-FPD, and GC-MS. J. Agric. Food Chem..

[32] Chen X.H., Chen D.J., Hai J., Sun H.Y., Chen Z., Hua Z., Li X.S., Fei Y., Chen C. (2019). Aroma characterization of Hanzhong black tea (*Camellia sinensis*) using solid phase extraction coupled with gas chromatography–mass spectrometry and olfactometry and sensory analysis. Food Chem..

[33] Wang S.Q., Chang Y., Liu B., Chen H.T., Sun B.G., Zhang N. (2021). Characterization of the key aroma-active compounds in Yongchuan Douchi (fermented soybean) by application of the sensomics approach. Molecules.

[34] Zhang J.L., Li J., Wang J., Sun B.G., Liu Y.P., Huang M.Q. (2021). Characterization of aroma-active compounds in Jasminum sambac concrete by aroma extract dilution analysis and odour activity value. Flavour Fragr. J..

[35] Rychlik M., Schieberle P.W., Grosch W. (1998). Compilation of Odor Thresholds, Odor Qualities and Retention Indices of Key Food Odorants.

[36] Qiu S., Chen K., Liu C., Wang Y.X., Chen T., Yan G.L., Li J.M. (2022). Non-saccharomyces yeasts highly contribute to characterisation of flavour profiles in greengage fermentation. Food Res. Int..

[37] Ma L.J., Gao M.M., Zhang L.Q., Qiao L., Li J.X., Du L.P., Zhang H.L., Wang H. (2022). Characterization of the key aroma-active compounds in high-grade Dianhong tea using GC-MS and GC-O combined with sensory-directed flavor analysis. Food Chem..

[38] Sanchez-Palomo E., Trujillo M., Ruiz A.G., González Viñas M.A. (2017). Aroma profile of malbec red wines from la mancha region: Chemical and sensory characterization. Food Res. Int..

[39] Du X.F., Finn C.E., Qian M.C. (2010). Volatile composition and odour-activity value of thornless ‘Black diamond’ and ‘Marion’ blackberries. Food Chem..

[40] Pang X.L., Yu W.S., Cao C.D., Yuan X.X., Qiu J., Kong F.Y., Wu J.H. (2019). Comparison of potent odorants in raw and ripened Pu-Erh tea infusions based on odor activity value calculation and multivariate analysis: Understanding the role of pile fermentation. J. Agric. Food Chem..

[41] Wang B., Meng Q., Xiao L., Li R.L., Peng C.H., Liao X.L., Yan J.N., Liu H.L., Xie G.H., Ho Q.T. (2022). Characterization of aroma compounds of pu-erh ripen tea using solvent assisted flavor evaporation coupled with gas chromatography-mass spectrometry and gas chromatography-olfactometry. Food Sci. Hum. Well..

