# Supplementary material for: Characterization of Key Odorants in Lingtou Dancong Oolong Tea and Their Differences Induced by Environmental Conditions from Different Altitudes
Source: Metabolites. 2022 Nov 3;12(11):1063. doi: 10.3390/metabo12111063 (PMC9695488; doi:10.3390/metabo12111063)
Supplement: Supplementary file 1 [file metabolites-12-01063-s001.zip › metabolites-1979767-SI.pdf]

Supplementary Materials

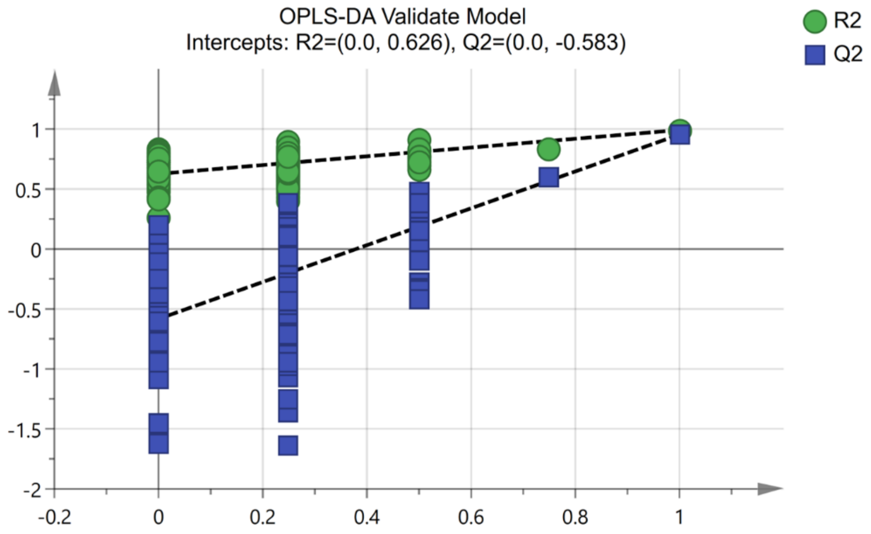

Figure S1. OPLS-DA model validation

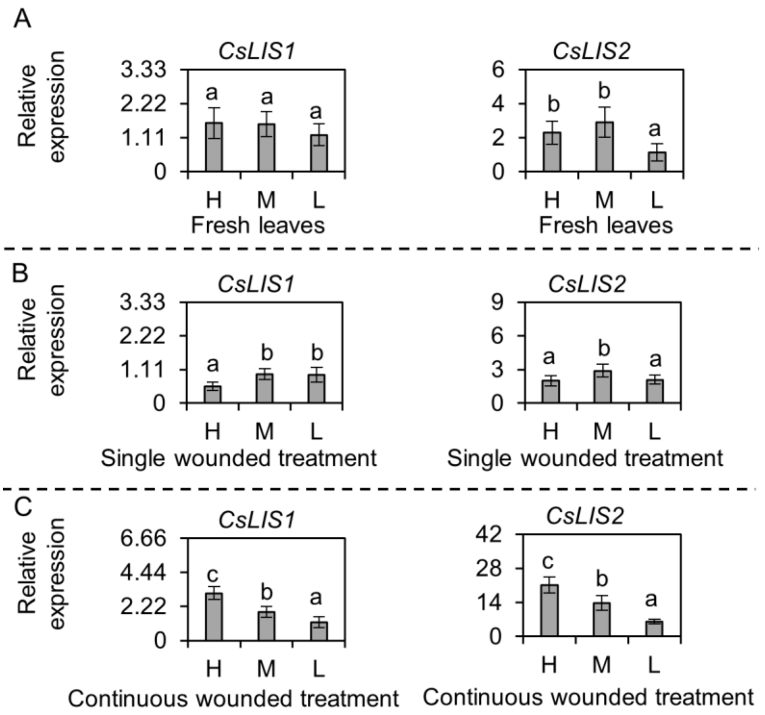

Figure S2. Expression level of *CsLISs* in fresh leaves and wounded leaves of Lingtou Dancong grown at different altitudes. (A) Expression levels of *CsLIS1* and *CsLIS2* in fresh leaves (*CsSAND* as reference gene); (B) Expression levels of *CsLIS1* and *CsLIS2* in leaves after single wounded treatment for 3 hours (*CsSAND* as reference gene); (C) Expression levels of *CsLIS1* and *CsLIS2* in leaves after continuous wounded treatment for 3 hours (*CsSAND* as reference gene). *LIS, linalool synthase*. Data are expressed as mean ± S. D. (n=3). Means distinguished with different letters are significantly different from each

other among high altitude, medium altitude and low altitude ($p \leq 0.05$). H, M and L represents the *C. sinensis cv.* Lingtou Dancong leaves picked from high, medium and low altitude respectively.

Table S1. Threshold information.

NO	Odorants	OT (μg/kg)	References
1	Butanal, 2-methyl-	1	[1]
2	Hexanal	4.5	[2]
3	Ethyl 2-methyl butyrate	n.f.	
4	2-Heptanone	140	[2]
5	Heptanal	550	[2]
6	(*Z*)-4-Heptenal	10	[2]
7	2,5-Dimethyl pyrazine	n.f.	
8	Diethyl disulfide	30	[3]
9	Dimethyl trisulfide	0.01	[4]
10	1-Octen-3-ol	1.5	[2]
11	Methyl heptenone	100	[5]
12	β-Myrcene	14	[6]
13	2-Pentyl furan	5.9	[2]
14	α-Phellandrene	n.f.	
15	(*Z*)-β-Ocimene	34	[7]
16	1-Ethyl-1H-pyrrole-2-carbaldehyde	37	[8]
17	(*E*)-2-Octenal	3	[2]
18	γ-Terpinene	55	[8]
19	3-Ethyl-2,5-dimethylpyrazine	8.6	[8]
20	Pyrazine, 2,6-diethyl-	n.f.	
21	Linalool	0.22	[9]
22	Nonanal	40	[2]
23	Hotrienol	110	[2]
24	2,6-Dimethyl-1,3,5,7-octatetraene, *E*, *E*-	n.f.	
25	5-Ethyl-6-methyl-3*E*-hepten-2-one	n.f.	
26	Nerol oxide	n.f.	
27	*trans*-Linalool oxide (pyranoid)	320	[2]
28	Terpinen-4-ol	4370	[10]
29	α-Terpineol	330	[2]
30	Methyl salicylate	40	[2]
31	(*E*, *E*)-2,4-Nonadienal	0.06	[11]
32	Nerol	300	[11]
33	*cis*-Citral	30	[6]

34	Geraniol	40	[2]
35	γ-Octanolactone	n.f.	
36	2-Phenyl-2-butenal	500	[10]
37	2,4-Decadienal	n.f.	
38	Indole	100	[2]
39	Theaspirane B	n.f.	
40	(*E*)-Methyl geranate	n.f.	
41	1,2-Dihydro-1,1,6-trimethylnaphthalene	n.f.	
42	γ-Nonanolactone	27	4
43	(*E*)-β-Damascenone	0.05	[7]
44	(*Z*)-Jasmone	21600	[5]
45	Dehydrodihydroionone	n.f.	
46	α-Ionone	76	[12]
47	Isoeugenol	n.f.	
48	γ-Decanolactone	n.f.	
49	trans-β-Ionone	7	[2]
50	*cis*-Jasmine lactone	7	[10]
51	δ-Dodecalactone	n.f.	
52	Dihydroactinidioide	500	[13]
53	(*E*)-Nerolidol	10	[14]
54	Hexyl benzoate	73	[10]
55	Caryophyllene oxide	n.f.	
56	Methyl jasmonate	3	[10]
57	α-Cadinol	n.f.	
58	Bisabolol Oxide B	n.f.	
59	*cis*-3-Hexenyl salicylate	115	[10]

OT, odor thresholds in water. The thresholds information and references in Table 1 are listed above. n.f., represents not found.

Table S2. The primers used for quantitative real time PCR (qRT-PCR) in the study.

Gene	Accession number	Forward primer 5'-3'	Reverse primer 5'-3'
CsEF-1α	KA280301.1	TTGGACAAGCTCAAGGCTGAACG	ATGGCCAGGAGCATCAAT GACAGT
CsSAND1	KM057790	TGAGTAACTCTGGCAAACCAATA	CCCATTCTCCACGAAGGAAA
CsLIS1	KF006849	GAGGGTTGATGAGTTTGTATG	TCACTTGGGTTCGTGTAAT
CsLIS2	KR873396	TTCAAGGACAAGGACGGAGGG	CCATGAACTTTGCCAGGCTCT

EF-1α, encoding elongation factor 1; SAND, SAND family protein; LIS, linalool synthase.

Table S3. Detailed information of Lingtou Dancong tea product at high altitude.

NO	Odorants	CAS	Description	RT (left)	RT (right)	Aroma Intensities (high altitude)					
						P1	P2	P3	P4	P5	P6
1	Butanal, 2-methyl-	96-17-3	Musty cocoa coffee nutty	4.19	4.53	2			2	1	
2	Hexanal	66-25-1	fresh green fatty aldehydic grass leafy fruity sweaty	6.75	6.85	2	3		2	2	
3	Ethyl 2-methyl butyrate	7452-79-1	sharp sweet green apple fruity	8.02	8.32	3	3		1	3	1
4	2-Heptanone	110-43-0	fruity spicy sweet herbal coconut woody	9.32	9.57	2				1	
5	Heptanal	111-71-7	fresh aldehydic fatty green herbal wine-lee ozone	9.65	9.80	3	3	1	4	2	1
6	(Z)-4-Heptenal	6728-31-0	fresh aldehydic fatty green herbal wine-lee ozone	9.65	9.80	3	3	1	4	2	1

7	2,5-Dimethyl pyrazine	123-32-0	peanut butter musty nutty woody roasted cocoa	9.92	10.18	2	3			2	
8	Diethyl disulfide	110-81-6	gassy ripe onion greasy garlic	10.30	10.50		3		3	2	2
9	Dimethyl trisulfide	3658-80-8	sulfurous cooked onion savory meaty	12.00	12.20	2	3		4	3	2
10	1-Octen-3-ol	3391-86-4	mushroom earthy green oily fungal raw chicken	12.20	12.40				2	4	2
11	Methyl heptenone	110-93-0	citrus green musty lemongrass apple	12.40	12.60				2	4	2
12	β-Myrcene	123-35-3	Peppery terpene spicy balsam plastic	12.60	12.70	3	3	1	4	4	2
13	2-Pentyl furan	3777-69-3	Fruity green earthy beany vegetable metallic	12.60	12.70	3	3	1	4	4	2
14	α-Phellandrene	99-83-2	citrus herbal terpene green woody peppery	13.10	13.42	3		3			2
15	(*Z*)-β-Ocimene	3338-55-4	warm floral herb flower sweet	14.70	14.90	3	4			2	2
16	1-Ethyl-1*H*-pyrrole-2-carbaldehyde	2167-14-8	burnt roasted smoky	14.89	15.02	2	3		1	3	1
17	(*E*)-2-Octenal	2548-87-0	fresh cucumber fatty green herbal banana waxy green leaf	15.12	15.40	3			3	3	
18	γ-Terpinene	99-85-4	oily woody terpene lemon/lime tropical herbal	15.02	15.21	3			3	3	
19	3-Ethyl-2,5-dimethylpyrazine	13360-65-1	potato cocoa roasted nutty	15.90	16.00	1	4		3		1
20	Pyrazine, 2,6-diethyl-	13067-27-1	nutty hazelnut	16.27	16.41	4	4	3	4	3	
21	Linalool	78-70-6	citrus floral sweet bois de rose woody green blueberry	16.48	16.84	3	4	2	4	3	1

22	Nonanal	124-19-6	waxy aldehydic rose fresh orris orange peel fatty peely	16.43	16.78	3	4	2	4	3	1
23	Hotrienol	29957-43-5	mouldy	16.97	17.17	3	3		4	4	2
24	2,6-Dimethyl-1,3,5,7-octatetraene, *E*, *E*-	460-01-5	Woody, herbal	17.76	17.88	3	2	2	1	4	2
25	5-Ethyl-6-methyl-3*E*-hepten-2-one	57283-79-1	Green, grassy, fresh	18.24	18.54	3	2	1	3	4	2
26	Nerol oxide	1786-08-9	green weedy cortex herbal diphenyl oxide narcissus celery	18.58	18.75	3	2	2	4	4	2
27	*trans*-Linalool oxide (pyranoid)	39028-58-5	Earthy, floral	19.23	19.46	2	3		3	4	1
28	Terpinen-4-ol	562-74-3	pepper woody earth musty sweet	19.47	19.72			2	3	4	2
29	α-Terpineol	98-55-5	pine terpene lilac citrus woody floral	19.80	20.04	3			3	2	1
30	Methyl salicylate	119-36-8	wintergreen mint	19.93	20.22	3	2	3	3	2	1
31	(*E*, *E*)-2,4-Nonadienal	5910-87-2	fatty melon waxy green violet leaf cucumber tropical fruit chicken fat	20.61	20.84	2			4	2	
32	Nerol	106-25-2	sweet natural neroli citrus magnolia	21.27	21.41		2	3			
33	*cis*-Citral	106-26-3	sweet citral lemon peel	21.62	21.76	3	3		4	3	2
34	Geraniol	106-24-1	sweet floral fruity rose waxy citrus	22.01	22.49	4	3	2	3		1
35	γ-Octanolactone	104-50-7	sweet coconut waxy creamy tonka dairy fatty	22.20	22.50	2			2	2	
36	2-Phenyl-2-butenal	4411-89-6	green, vegetative, floral, cocoa and nutty	22.63	23.11	2			3	3	1
37	2,4-Decadienal		orange sweet fresh citrus fatty green	23.23	23.54	3	3		2	2	
38	Indole	120-72-9	animal floral moth ball fecal naphthelene	23.77	24.08	3	4	1	2	2	2

39	Theaspirane B	36431-72-8	tea herbal green wet tobacco leaf metallic woody spicy	24.11	24.24	2			3		2
40	(*E*)-Methyl geranate	1189-09-9	waxy green fruity flower	24.29	24.69	3			4	2	
41	1,2-Dihydro-1,1,6-trimethylnaphthalene	30364-38-6	licorice	25.46	25.60	4	3	1	2		
42	γ-Nonanolactone	104-61-0	coconut creamy waxy sweet buttery oily	25.67	25.87	3		2	3	2	1
43	(*E*)-β-Damascenone	23726-93-4	apple rose honey tobacco sweet	26.12	26.50	2	3	2	2	3	1
44	(*Z*)-Jasmone	488-10-8	woody herbal floral spicy jasmine celery	26.69	27.11	3	3	2	2	3	1
45	Dehydrodihydroionone	20483-36-7	floral	27.46	27.67	3	4		2	3	
46	α-Ionone	127-41-3	sweet woody floral violet orris tropical fruity	27.69	28.07	3	4	1	2	4	2
47	Isoeugenol	5932-68-3	sweet spicy carnation phenolic floral	28.52	28.88	3		1	3	4	1
48	γ-Decanolactone	706-14-9	fresh oily waxy peach coconut buttery sweet	28.99	29.19	3			2	4	
49	*trans*-β-Ionone	79-77-6	dry powdery floral woody orris	29.40	29.87	4	4	3	4	3	1
50	*cis*-Jasmine lactone	25524-95-2、100428-67-9	creamy waxy jasmine peach coconut	29.97	30.23	3	4	4	4	3	
51	δ-Dodecalactone	705-86-2	fresh sweet oily coconut fruity peach creamy dairy	29.97	30.23	3	4	4	4	3	
52	Dihydroactinidioide	15356-74-8	ripe apricot red fruit woody	30.76	31.08	2	3	4	2	2	2
53	(*E*)-Nerolidol	40716-66-3	floral green citrus woody waxy	31.70	31.90				1	2	
54	Hexyl benzoate	6789-88-4	fresh balsam sappy clean woody	32.12	32.32	1		2	1	2	
55	Caryophyllene oxide	1139-30-6	sweet fresh dry woody spicy	32.56	32.93	2					
56	Methyl jasmonate	1211-29-6	floral fresh petal magnolia oily waxy	34.16	34.32	2		1			

57	*α*-Cadinol	481-34-5	herb wood	34.38	34.70	3	3	4	2	2	2
58	Bisabolol oxide B	26184-88-3	Woody, herbal	34.38	34.70	3	3	4	2	2	2
59	*cis*-3-Hexenyl salicylate	65405-77-8	floral green metallic herbal balsam	34.95	35.25	3	2		2		1

RT, retention time. 1-4 represents the intensity of the aroma, 1 indicates the lowest intensity, 4 indicates the highest intensity. P1-P6 represents different evaluator. During the extraction, 1 g tea powder was brewed in 10 mL water (100°C) for GC-O/MS analysis. Qualitative analysis was performed by a combination of NIST14 database comparisons, RI values and olfactive evaluation.

Table S4. Detailed information of Lingtou Dancong tea product at medium altitude.

NO	Odorants	CAS	Description	RT (left)	RT (right)	Aroma Intensities (medium altitude)					
						P1	P2	P3	P4	P5	P6
1	Butanal, 2-methyl-	96-17-3	Musty cocoa coffee nutty	4.22	4.41	2			1	1	
2	Hexanal	66-25-1	fresh green fatty aldehydic grass leafy fruity sweaty	6.74	6.91	2	3		1	2	2
3	Ethyl 2-methyl butyrate	7452-79-1	sharp sweet green apple fruity	8.02	8.38	3	3		1	2	
4	2-Heptanone	110-43-0	fruity spicy sweet herbal coconut woody	9.32	9.57						
5	Heptanal	111-71-7	fresh aldehydic fatty green herbal wine-lee ozone	9.61	9.98	3	3		4	2	1
6	(*Z*)-4-Heptenal	6728-31-0	fresh aldehydic fatty green herbal wine-lee ozone	9.57	9.61	3	3		4	2	1
7	2,5-Dimethyl pyrazine	123-32-0	peanut butter musty nutty woody roasted cocoa	9.92	10.18	3	3		4	2	1
8	Diethyl disulfide	110-81-6	gassy ripe onion greasy garlic	10.40	10.76		2		1	1	2

9	Dimethyl trisulfide	3658-80-8	sulfurous cooked onion savory meaty	12.00	12.20	2	2	3	2	3	
10	1-Octen-3-ol	3391-86-4	mushroom earthy green oily fungal raw chicken	12.20	12.40	3			2	4	
11	Methyl heptenone	110-93-0	citrus green musty lemongrass apple	12.40	12.60	3	3	2	4	4	2
12	β-Myrcene	123-35-3	Peppery terpene spicy balsam plastic	12.60	12.70	3	3	2	4	4	2
13	2-Pentyl furan	3777-69-3	Fruity green earthy beany vegetable metallic	12.60	12.70	3	3	2	4	4	2
14	α-Phellandrene	99-83-2	citrus herbal terpene green woody peppery	12.98	13.21	3	2		3	2	
15	(*Z*)-β-Ocimene	3338-55-4	warm floral herb flower sweet	14.71	14.86	3	3		1	2	2
16	1-Ethyl-1*H*-pyrrole-2-carbaldehyde	2167-14-8	burnt roasted smoky	14.89	15.02	2		1	4	3	2
17	(*E*)-2-Octenal	2548-87-0	fresh cucumber fatty green herbal banana waxy green leaf	15.12	15.40	3			2	4	
18	γ-Terpinene	99-85-4	oily woody terpene lemon/lime tropical herbal	15.04	15.22	3			2	4	
19	3-Ethyl-2,5-dimethylpyrazine	13360-65-1	potato cocoa roasted nutty	15.90	16.00	2	2	1	2		2
20	Pyrazine, 2,6-diethyl-	13067-27-1	nutty hazelnut	16.08	16.38	3	3	3	3	3	
21	Linalool	78-70-6	citrus floral sweet bois de rose woody green blueberry	16.41	16.75	3	4		3	3	3
22	Nonanal	124-19-6	waxy aldehydic rose fresh orris orange peel fatty peely	16.43	16.78	3	4		3	3	3
23	Hotrienol	29957-43-5	mouldy	16.85	17.05	2		3	4	4	3

24	2,6-Dimethyl-1,3,5,7-octatetraene, *E*, *E*-	460-01-5	Woody, herbal	17.76	17.88	3	2	1	2	4	2
25	5-Ethyl-6-methyl-3*E*-hepten-2-one	57283-79-1	Green, grassy, fresh	18.24	18.54	2	3		3	4	1
26	Nerol oxide	1786-08-9	green weedy cortex herbal diphenyl oxide narcissus celery	18.58	18.75	3	4	2	4	4	1
27	*trans*-Linalool oxide (pyranoid)	39028-58-5	Earthy, floral	19.20	19.43		2	2	3	3	
28	Terpinen-4-ol	562-74-3	pepper woody earth musty sweet	19.49	19.65	3	2	2	3	3	
29	α-Terpineol	98-55-5	pine terpene lilac citrus woody floral	19.80	20.04	2			2	2	
30	Methyl salicylate	119-36-8	wintergreen mint	20.13	20.25	4	3	2	2	3	1
31	(*E*, *E*)-2,4-Nonadienal	5910-87-2	fatty melon waxy green violet leaf cucumber tropical fruit chicken fat	20.61	20.84	3	3		3	4	2
32	Nerol	106-25-2	sweet natural neroli citrus magnolia	21.27	21.41	2	2				4
33	*cis*-Citral	106-26-3	sweet citral lemon peel	21.62	21.88	3	3	3	2	3	1
34	Geraniol	106-24-1	sweet floral fruity rose waxy citrus	21.99	22.35	3	2	1	1	3	2
35	γ-Octanolactone	104-50-7	sweet coconut waxy creamy tonka dairy fatty	22.36	22.54	2	2			3	
36	2-Phenyl-2-butenal	4411-89-6	green, vegetative, floral, cocoa and nutty	22.65	22.79	3	2		1		1
37	2,4-Decadienal		orange sweet fresh citrus fatty green	23.20	23.52	2		1	1		
38	Indole	120-72-9	animal floral moth ball fecal naphthelene	23.80	23.96	4	4		1	2	
39	Theaspirane B	36431-72-8	tea herbal green wet tobacco leaf metallic woody spicy	24.13	24.23	3	4		3	3	2
40	(*E*)-Methyl geranate	1189-09-9	waxy green fruity flower	24.29	24.52	4	4		3	4	

41	1,2-Dihydro-1,1,6-trimethylnaphthalene	30364-38-6	licorice	25.46	25.60	2		3	1		
42	γ-Nonanolactone	104-61-0	coconut creamy waxy sweet buttery oily	25.61	25.72	3		3	1	3	
43	(*E*)-β-Damascenone	23726-93-4	apple rose honey tobacco sweet	26.32	26.54	2	3	2	1	4	1
44	(*Z*)-Jasmone	488-10-8	woody herbal floral spicy jasmine celery	26.61	27.11	2	3	2	1	4	
45	Dehydrodihydroionone	20483-36-7	floral	27.33	27.68			2	2	2	
46	α-Ionone	127-41-3	sweet woody floral violet orris tropical fruity	27.65	27.76	3	4	2	2	4	
47	Isoeugenol	5932-68-3	sweet spicy carnation phenolic floral	28.46	28.61	2	3	2	2	3	
48	γ-Decanolactone	706-14-9	fresh oily waxy peach coconut buttery sweet	28.99	29.19	2	3	3	1	3	
49	*trans*-β-Ionone	79-77-6	dry powdery floral woody orris	29.37	29.68	3	4	2	2	4	2
50	*cis*-Jasmine lactone	25524-95-2、100428-67-9	creamy waxy jasmine peach coconut	29.97	30.23	2	3	3	1	4	
51	δ-Dodecalactone	705-86-2	fresh sweet oily coconut fruity peach creamy dairy	29.97	30.23	2	3	3	1	4	
52	Dihydroactinidioide	15356-74-8	ripe apricot red fruit woody	30.76	31.10	3	2	2		4	
53	(*E*)-Nerolidol	40716-66-3	floral green citrus woody waxy	31.70	31.90						
54	Hexyl benzoate	6789-88-4	fresh balsam sappy clean woody	31.94	32.11	2	3	1			
55	Caryophyllene oxide	1139-30-6	sweet fresh dry woody spicy	32.56	32.93	2	2	1	1	2	
56	Methyl jasmonate	1211-29-6	floral fresh petal magnolia oily waxy	34.16	34.32						
57	α-Cadinol	481-34-5	herb wood	34.38	34.70	3	3	3	2	3	1
58	Bisabolol oxide B	26184-88-3	Woody, herbal	34.38	34.70	3	3	3	2	3	1
59	*cis*-3-Hexenyl salicylate	65405-77-8	floral green metallic herbal balsam	34.95	35.25	3	2		4		

RT, retention time. 1-4 represents the intensity of the aroma, 1 indicates the lowest intensity, 4 indicates the highest intensity. P1-P6 represents different evaluator. During the extraction, 1 g tea powder was brewed in 10 mL water (100°C) for GC-O/MS analysis. Qualitative analysis was performed by a combination of NIST14 database comparisons, RI values and olfactive evaluation.

Table S5. Detailed information of Lingtou Dancong tea product at low altitude.

NO	Odorants	CAS	Description	RT (left)	RT (right)	Aroma Intensities (low altitude)					
						P1	P2	P3	P4	P5	P6
1	Butanal, 2-methyl-	96-17-3	Musty cocoa coffee nutty	4.22	4.41	1	1		1		
2	Hexanal	66-25-1	fresh green fatty aldehydic grass leafy fruity sweaty	6.71	6.91	1	2	1	1		
3	Ethyl 2-methyl butyrate	7452-79-1	sharp sweet green apple fruity	8.02	8.38	3	2	1	2	1	2
4	2-Heptanone	110-43-0	fruity spicy sweet herbal coconut woody	9.32	9.57						
5	Heptanal	111-71-7	fresh aldehydic fatty green herbal wine-lee ozone	9.57	9.61	3	3	4	2		1
6	(Z)-4-Heptenal	6728-31-0	fresh aldehydic fatty green herbal wine-lee ozone	9.57	9.61	3	2	4	2	2	1
7	2,5-Dimethyl pyrazine	123-32-0	peanut butter musty nutty woody roasted cocoa	9.94	10.34	2	2	3			
8	Diethyl disulfide	110-81-6	gassy ripe onion greasy garlic	10.37	10.47	2		3			3
9	Dimethyl trisulfide	3658-80-8	sulfurous cooked onion savory meaty	12.03	12.20	2	3	2	3	2	2
10	1-Octen-3-ol	3391-86-4	mushroom earthy green oily fungal raw chicken	12.20	12.40	3		3	4		2
11	Methyl heptenone	110-93-0	citrus green musty lemongrass apple	12.40	12.60	3		2	4		3

12	β-Myrcene	123-35-3	Peppery terpene spicy balsam plastic	12.60	12.70	3	3		4	2	
13	2-Pentyl furan	3777-69-3	Fruity green earthy beany vegetable metallic	12.60	12.70	3	3		4	2	
14	α-Phellandrene	99-83-2	citrus herbal terpene green woody peppery	12.95	13.29	3		3	2		
15	(*Z*)-β-Ocimene	3338-55-4	warm floral herb flower sweet	14.68	14.84	4	4	3	2		3
16	1-Ethyl-1*H*-pyrrole-2-carbaldehyde	2167-14-8	burnt roasted smoky	14.86	14.96	4		4	4	2	2
17	(*E*)-2-Octenal	2548-87-0	fresh cucumber fatty green herbal banana waxy green leaf	15.12	15.40	2	4	3	4	3	
18	γ-Terpinene	99-85-4	oily woody terpene lemon/lime tropical herbal	15.12	15.20	2	4	3	4	3	
19	3-Ethyl-2,5-dimethylpyrazine	13360-65-1	potato cocoa roasted nutty	15.85	16.08	4	4	4			3
20	Pyrazine, 2,6-diethyl-	13067-27-1	nutty hazelnut	16.08	16.38	2	4	4	4	3	
21	Linalool	78-70-6	citrus floral sweet / woody green blueberry	16.79	16.89	3	3	4	4	2	4
22	Nonanal	124-19-6	waxy aldehydic rose fresh orris orange peel fatty peely	16.43	16.78	2		3			3
23	Hotrienol	29957-43-5	mouldy	17.05	17.24	2	3		3	2	4
24	2,6-Dimethyl-1,3,5,7-octatetraene, *E*, *E*-	460-01-5	Woody, herbal	17.72	17.78	3	2	2	4	1	3
25	5-Ethyl-6-methyl-3*E*-hepten-2-one	57283-79-1	Green, grassy, fresh	18.24	18.54	3	3	2	4	1	1
26	Nerol oxide	1786-08-9	green weedy cortex herbal diphenyl oxide narcissus celery	18.58	18.75	3	3	4	4	3	3

27	*trans*-Linalool oxide (pyranoid)	39028-58-5	Earthy, floral	19.30	19.43	2	2	4	4	3	3
28	Terpinen-4-ol	562-74-3	pepper woody earth musty sweet	19.49	19.65	2	3	4	4		
29	α-Terpineol	98-55-5	pine terpene lilac citrus woody floral	19.79	20.09	2	2	3	4		
30	Methyl salicylate	119-36-8	wintergreen mint	20.13	20.38	2		3	3	1	3
31	(*E*, *E*)-2,4-Nonadienal	5910-87-2	fatty melon waxy green violet leaf cucumber tropical fruit chicken fat	20.68	20.90	3	4	4	4		2
32	Nerol	106-25-2	sweet natural neroli citrus magnolia	21.27	21.41	2	4	2	2	1	1
33	*cis*-Citral	106-26-3	sweet citral lemon peel	21.62	21.78	2		4	3	2	3
34	Geraniol	106-24-1	sweet floral fruity rose waxy citrus	21.99	22.35	3	2	2	2	3	3
35	γ-Octanolactone	104-50-7	sweet coconut waxy creamy tonka dairy fatty	22.36	22.54	2	3	3	2	3	
36	2-Phenyl-2-butenal	4411-89-6	green, vegetative, floral, cocoa and nutty	22.65	22.79	2		2	3		3
37	2,4-Decadienal		orange sweet fresh citrus fatty green	23.20	23.52	2		2	4	2	
38	Indole	120-72-9	animal floral moth ball fecal naphthelene	23.80	23.96	4	4	3	4	2	2
39	Theaspirane B	36431-72-8	tea herbal green wet tobacco leaf metallic woody spicy	24.13	24.23	4	3	4	4	2	3
40	(*E*)-Methyl geranate	1189-09-9	waxy green fruity flower	24.26	24.41	2	3	3	3	2	3
41	1,2-Dihydro-1,1,6-trimethylnaphthalene	30364-38-6	licorice	25.07	25.42	2	3	2	2		
42	γ-Nonanolactone	104-61-0	coconut creamy waxy sweet buttery oily	25.71	25.81	3		3	3	3	
43	(*E*)-β-Damascenone	23726-93-4	apple rose honey tobacco sweet	26.24	26.49	2	3	3	4	3	3

44	(*Z*)-Jasmone	488-10-8	woody herbal floral spicy jasmine celery	26.88	27.01	3	3	3	2	2	2
45	Dehydrodihydroionone	20483-36-7	floral	27.48	27.64	1	3	3	3	2	
46	*α*-Ionone	127-41-3	sweet woody floral violet orris tropical fruity	27.65	27.95	2	3	3	4	2	2
47	Isoeugenol	5932-68-3	sweet spicy carnation phenolic floral	28.46	28.84	2	2	3	2	3	1
48	γ-Decanolactone	706-14-9	fresh oily waxy peach coconut buttery sweet	28.93	29.14	3		3		3	1
49	*trans*-β-Ionone	79-77-6	dry powdery floral woody orris	29.34	29.69	4	4	4	4	2	3
50	*cis*-Jasmine lactone	25524-95-2、100428-67-9	creamy waxy jasmine peach coconut	29.79	30.06	4	3	4	4	3	1
51	δ-Dodecalactone	705-86-2	fresh sweet oily coconut fruity peach creamy dairy	29.97	30.23	2	3	1	4	3	
52	Dihydroactinidioide	15356-74-8	ripe apricot red fruit woody	30.76	31.10	3	2		2		2
53	(*E*)-Nerolidol	40716-66-3	floral green citrus woody waxy	31.70	31.90				2	1	
54	Hexyl benzoate	6789-88-4	fresh balsam sappy clean woody	32.11	32.28	2		1	2	2	2
55	Caryophyllene oxide	1139-30-6	sweet fresh dry woody spicy	32.56	32.93	2	2		2	2	1
56	Methyl jasmonate	1211-29-6	floral fresh petal magnolia oily waxy	34.16	34.32	2		2	2		2
57	*α*-Cadinol	481-34-5	herb wood	34.38	34.70	3	3	3	4	3	4
58	Bisabolol oxide B	26184-88-3	Woody, herbal	34.38	34.70	3	3	3	4	3	4
59	*cis*-3-Hexenyl salicylate	65405-77-8	floral green metallic herbal balsam	34.95	35.25	2	2	2	3	2	3

RT, retention time. 1-4 represents the intensity of the aroma, 1 indicates the lowest intensity, 4 indicates the highest intensity. P1-P6 represents different evaluator. During the extraction, 1 g tea powder was brewed in 10 mL water (100°C) for GC-O/MS analysis. Qualitative analysis was performed by a combination of NIST14 database comparisons, RI values and olfactive evaluation.

Reference

1. Wang, M.Q.; Ma, W.J.; Shi, J.; Zhu, Y.; Lin, Z.; Lv, H.P. Characterization of the key aroma compounds in Longjing tea using stir bar sorptive extraction (SBSE) combined with gas chromatography-mass spectrometry (GC-MS), gas chromatography-olfactometry (GC-O), odor activity value (OAV), and aroma recombination. *Food Res. Int.* **2020**, 130, 108908. https://doi.org/10.1016/j.foodres.2019.108908.
2. Zhu, J.C.; Chen, F.; Wang, L.Y.; Niu, Y.W.; Yu, D.; Shu, C.; Chen, H.X.; Wang, H.L.; Xiao, Z.B. Comparison of aroma-active volatiles in oolong tea infusions using GC-Olfactometry, GC-FPD, and GC-MS. *J. Agr. Food Chem.* **2015**, 63, 7499-7510. https://doi.org/10.1021/acs.jafc.5b02358.
3. Chen, X.H.; Chen, D.J.; Hai, J.; Sun, H.Y.; Chen, Z.; Hua, Z.; Li, X.S.; Fei, Y., Chen, C. Aroma characterization of Hanzhong black tea (*Camellia sinensis*) using solid phase extraction coupled with gas chromatography–mass spectrometry and olfactometry and sensory analysis. *Food Chem.* **2019**, 274, 130-136. https://doi.org/10.1016/j.foodchem.2018.08.124.
4. Wang, S.Q.; Chang, Y.; Liu, B.; Chen, H.T.; Sun, B.G.; Zhang, N. Characterization of the key aroma-active compounds in Yongchuan Douchi (fermented soybean) by application of the sensomics approach. *Molecules* **2021**, 26, 3048. https://doi.org/10.3390/molecules26103048.
5. Zhang, J.L.; Li, J.; Wang, J.; Sun, B.G.; Liu, Y.P.; Huang, M.Q. Characterization of aroma-active compounds in Jasminum sambac concrete by aroma extract dilution analysis and odour activity value. *Flavour Fragr. J.* **2021**, 36,197-206. https://doi.org/10.1002/ffj.3631.
6. Rychlik, M.; Schieberle, P. W.; Grosch, W. Compilation of odor thresholds, odor qualities and retention indices of key food odorants. Deutsche Forschungsanstalt fur Lebensmittelchemie and Institut fur Lebensmittelchemie der Technischen Universitat Munchen, Garching, Germany, 1998.
7. Qiu, S.; Chen, K.; Liu, C.; Wang, Y.X.; Chen, T.; Yan, G.L.; Li, J.M. Non-saccharomyces yeasts highly contribute to characterisation of flavour profiles in greengage fermentation. *Food Res. Int*. 2022, **157**, 111391. https://doi.org/10.1016/j.foodres.2022.111391.
8. Ma, L.J.; Gao, M.M.; Zhang, L.Q.; Qiao, L.; Li, J.X.; Du, L.P., Zhang, H.L.; Wang, H. Characterization of the key aroma-active compounds in high-grade Dianhong tea using GC-MS and GC-O combined with sensory-directed flavor analysis. *Food Chem.* **2022**, 378, 132058. https://doi.org/10.1016/j.foodchem.2022.132058.
9. Sanchez-Palomo, E.; Trujillo, M.; Ruiz, A.G.; González Viñas, M.A. Aroma profile of malbec red wines from la mancha region: Chemical and sensory characterization. *Food Res. Int.* 2017, **100**, 201-208. https://doi.org/10.1016/j.foodres.2017.06.036.
10. Guo, X.Y.; Schwab, W.; Ho, C.T.; Song, C.K.; Wan, X.C. Characterization of the aroma profiles of oolong tea made from three tea cultivars by both GC-MS and GC-IMS. *Food Chem.* 2022, **376**, 131933. https://doi.org/10.1016/j.foodchem.2021.131933.
11. Zhai, X.T.; Zhang, L.; Granvogl, M.; Ho, Q.T.; Wan, X.C. Flavor of tea (*Camellia sinensis*): A review on odorants and analytical techniques. *Compr. Rev. Food Sci. F.* **2022**, 21, 3867-3909. https://doi.org/10.1111/1541-4337.12999.
12. Du, X.F.; Finn, C.E.; Qian; M.C. Volatile composition and odour-activity value of thornless 'Black diamond' and 'Marion' blackberries. *Food Chem.* **2010**, 119, 1127-1134. https://doi.org/10.1016/j.foodchem.2009.08.024.
13. Pang, X.L.; Yu, W.S.; Cao, C.D.; Yuan, X.X.; Qiu, J., Kong, F.Y.; Wu, J.H. Comparison of potent odorants in raw and ripened Pu-Erh tea infusions based on odor activity value calculation and multivariate analysis: understanding the role of pile fermentation. *J. Agric. Food Chem.* **2019**, 67, 13139-13149 https://doi.org/10.1021/acs.jafc.9b05321.
14. Wang, B.; Meng, Q.; Xiao, L.; Li, R.L.; Peng, C.H.; Liao, X.L., Yan, J.N., Liu, H.L.; Xie, G.H.; Ho, Q.T., et al. Characterization of aroma compounds of pu-erh ripen tea using solvent assisted flavor evaporation coupled with gas chromatography-mass spectrometry and gas chromatography-olfactometry. *Food Sci. Hum. Well.* **2022**, 11, 618-626. https://doi.org/10.1016/j.fshw.2021.12.018.
